# Ligand-conjugated multiwalled carbon nanotubes for cancer targeted drug delivery

**DOI:** 10.3389/fphar.2024.1417399

**Published:** 2024-07-25

**Authors:** Chanchal Kiran Thakur, Chandrabose Karthikeyan, Charles R. Ashby, Rabin Neupane, Vishal Singh, R. Jayachandra Babu, N. S. Hari Narayana Moorthy, Amit K. Tiwari

**Affiliations:** ^1^ Cancept Therapeutics Laboratory, Department of Pharmacy, Indira Gandhi National Tribal University, Lalpur, India; ^2^ Chhattrapati Shivaji Institute of Pharmacy, Durg, Chhattisgarh, India; ^3^ Department of Pharmaceutical Sciences, College of Pharmacy, St. John’s University, Queens, NY, United States; ^4^ Department of Pharmacology and Experimental Therapeutics, College of Pharmacy and Pharmaceutical Sciences, University of Toledo, Toledo, OH, United States; ^5^ Department of Nutrition, State College, Pennsylvania State University, University Park, PA, United States; ^6^ Department of Drug Discovery and Development, Harrison School of Pharmacy, Auburn University, Auburn, AL, United States; ^7^ Department of Pharmaceutical Sciences, College of Pharmacy, University of Arkansas for Medical Sciences, Little Rock, AR, United States

**Keywords:** multiwalled carbon nanotubes, cancer, vitamins, targeted drug delivery, anticancer drugs, cytotoxicity

## Abstract

Multiwalled carbon nanotubes (MWCNTs) are at the forefront of nanotechnology-based advancements in cancer therapy, particularly in the field of targeted drug delivery. The nanotubes are characterized by their concentric graphene layers, which give them outstanding structural strength. They can deliver substantial doses of therapeutic agents, potentially reducing treatment frequency and improving patient compliance. MWCNTs’ diminutive size and modifiable surface enable them to have a high drug loading capacity and penetrate biological barriers. As a result of the extensive research on these nanomaterials, they have been studied extensively as synthetic and chemically functionalized molecules, which can be combined with various ligands (such as folic acid, antibodies, peptides, mannose, galactose, polymers) and linkers, and to deliver anticancer drugs, including but not limited to paclitaxel, docetaxel, cisplatin, doxorubicin, tamoxifen, methotrexate, quercetin and others, to cancer cells. This functionalization facilitates selective targeting of cancer cells, as these ligands bind to specific receptors overexpressed in tumor cells. By sparing non-cancerous cells and delivering the therapeutic payload precisely to cancer cells, this therapeutic payload delivery ability reduces chemotherapy systemic toxicity. There is great potential for MWCNTs to be used as targeted delivery systems for drugs. In this review, we discuss techniques for functionalizing and conjugating MWCNTs to drugs using natural and biomacromolecular linkers, which can bind to the cancer cells’ receptors/biomolecules. Using MWCNTs to administer cancer drugs is a transformative approach to cancer treatment that combines nanotechnology and pharmacotherapy. It is an exciting and rich field of research to explore and optimize MWCNTs for drug delivery purposes, which could result in significant benefits for cancer patients.

## 1 Introduction

Cancer continues to be a major global health concern, with 20 million new cases and 9.7 million deaths in 2022. This situation is not expected to improve, as projected statistics highlight a significant rise in the number of new cancer cases (29.9 million) and mortality (15.3 million) by the year 2040 (Bray et al., 2024).

Chemotherapeutic agents, e.g., Cisplatin, Docetaxel, Doxorubicin, Fluorouracil, Gemcitabine, Methotrexate, and Paclitaxel are the mainstay of therapeutic regimens for several types of cancer (Raza et al., 2016; Thakur et al., 2016). Still, dose-dependent adverse and toxic effects, low aqueous solubility, poor tissue specificity, low stability, short half-lives, and low tissue penetration seriously limit their clinical use (Dy and Adjei, 2013; Tran et al., 2019; Rele et al., 2024). Nanocarrier-based drug delivery systems such as liposomes (e.g., polymeric liposomes and magneto-liposomes), nanoparticles (e.g., magnetic nanoparticles and polymeric nanoparticles), and advanced technology polymers (e.g., dendrimers and carbon nanotubes) can overcome these limitations by entrapping and delivering the drug in the cancer tissue (Mainarden et al., 2004; Yun et al., 2015; Reza et al., 2018).

Among the different classes of nanocarrier-based drug delivery systems, carbon nanotubes (CNTs) represent a novel class of nanocarriers that offers numerous advantages such as a high drug loading capacity, large surface area, sustained drug release, and selective targeting potential (Pate et al., 2019; Thakur et al., 2023).

**FIGURE 1:**
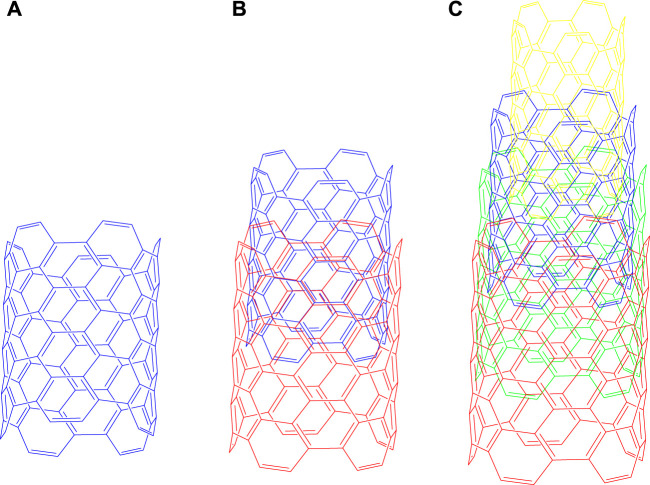
Types of Carbon Nanotubes **(A)** Single - walled, **(B)** Double - walled, **(C)** Multi - walled carbon nanotubes. Single-walled carbon nanotubes consist of only one layer of graphene, which forms six-atom carbon rings in a hexagonal shape, whereas the multilayer nanotubes consist of concentric layers of graphene in cylindrical shape.

Currently, CNTs, with unidimensional and graphitic hexagonal structure, containing horizontally arranged benzene rings, are being developed (Kesharwani et al., 2012; Mehra et al., 2015). Clinically, CNTs have been used based on their high aspect ratios, diminutively minuscule sizes, significant voluminous surface areas, facile perforate membrane and their conjugation or encapsulation with numerous therapeutic molecules (Patole et al., 2008; Kesharwani et al., 2012; Rele et al., 2024). Overall, CNTs represent an important biomaterial that can be used for the distribution of biomolecules and drugs, that can be generally classified into the following groups, according to their sizes and construction or arrangement, as single walled, double walled or multiwalled carbon nanotubes (MWCNTs), as shown in Figure 1.

### 1.1 Single-walled carbon nanotubes (SWCNTs)

MWCNTs consist of a graphene unilayer sheet that is wrapped into a cylindrical tube. MWCNTs can be further classified as zigzag, armchair and chiral (Cherukuri et al., 2006). Its diameter is approximately 1 nm and the length of tube is long because of the geometric arrangement of the nanotubes (Murakami et al., 2004; Jha et al., 2020).

### 1.2 Double - walled carbon nanotubes (DWCNTs)

DWCNTs consist of two layers of a cylindrically arranged graphite sheet. The structure and properties of DWCNTs are comparable to MWCNTs; however, the outer wall of the DWCNTs is a coaxial nanostructure that contains a two dimensional graphene sheet rolled into a cylindrical form (Jha et al., 2020). The cylindrical tubes that are less than 1 nm in diameter are not considered to be DWCNTs (Tison et al., 2008).

### 1.3 Multiwalled - carbon nanotubes (MWCNTs)

Currently, MWCNTs are nanotubes that are being used to develop effective nanomedicines (Srivastava et al., 2011; Garcia et al., 2015; Singh et al., 2016; Martins et al., 2023). MWCNTs offer several advantages over SWCNTs and DWCNTs, including higher drug loading capacity, larger surface area, and multiple sites for ligand, linker, and drug conjugation. MWCNTs provide a sustained release and a greater selective targeting potential that decreases toxicity and increases metabolic stability, with a more rigid and higher aspect ratio 1:1000 diameter/length ratio and a decrease the leakage of loaded drugs due to the presence of multiple layers nanomedicines (Tison et al., 2008; Kayat et al., 2011; Prasek et al., 2011; Mehra et al., 2016). Furthermore, fabricated/modified/conjugated MWCNTs have been used as biomarkers in cancer cells and as imaging and cancer diagnostic compounds nanomedicines (Ji et al., 2010; Wang et al., 2015). Many reviews published in this area predominantly focus on the use of carbon nanotubes for drug-delivery nanomedicines (Ethissi et al., 2012; Kushwaha et al., 2013; Son et al., 2016; Sanginario et al., 2017). In this review, we focus on the functionalization and ligand conjugation strategies of MWCNTs concerning their applications in the delivery of drugs to cancer cells.

#### 1.3.1 The structure of MWCNTs

MWCNTs consist of 3–10 layers of concentric tubes of graphene that interact with one another by van der Waals (vdW) forces (Figure 1), with each layer being separated from one another by approximately 0.34 nm, a distance that is greater than that of the inner layer sheets nanomedicines (Kulkarni et al., 2010; Lehman et al., 2011; Sahebian et al., 2016; Martins et al., 2023). The structures of the MWCNTs have been explained by the Russian doll model (RDM) and parchment model (PM) nanomedicines (Das et al., 2015a). In the RDM, the graphite sheets are arranged in cylinders, whereas in the PM, the graphite sheets are rolled into one sheet in the region, similar to a newspaper (Yang et al., 2007). MWCNTs have been reported to target, release and distribute drugs in various tissues, including cancer cells (Das et al., 2015b). Consequently, MWCNTS are used as nanocarriers for anticancer drug delivery and therapy to a targeted site in a sustained manner (Froy et al., 1999; Raza et al., 2016). The pristine MWCNTs are not appropriate for interaction with biomolecules or bioactive compounds, as they typically form bundles or aggregates that poorly disperse in aqueous solution (Yang et al., 2007; Sahoo et al., 2011). This problem could be circumvented by functionalization, which is one of the methods used to improve the dispersion and solubility of MWCNTs. For example, the modification of the surface of MWCNTs with various chemical groups decreases the likelihood of aggregation, which can decrease toxicity (Froy et al., 1999; Raza et al., 2016; Sheikhpour et al., 2017).

#### 1.3.2 Advances in the functionalization of MWCNTs

**FIGURE 2:**
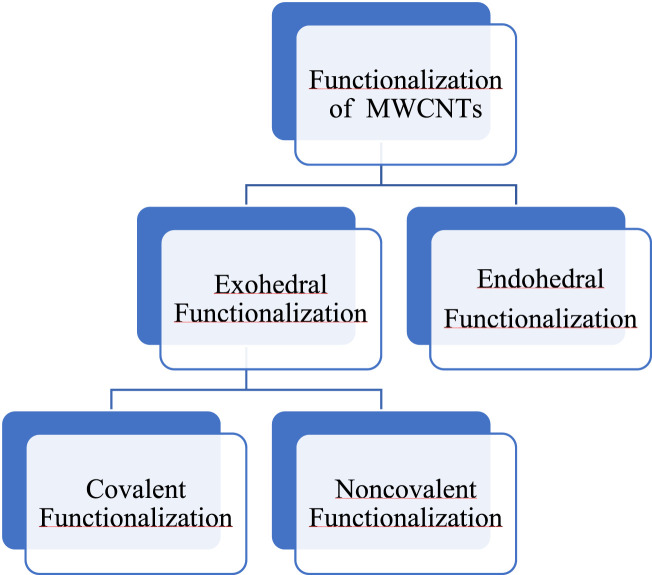
Chemical methods for the functionalization of MWCNTs.

**FIGURE 3:**
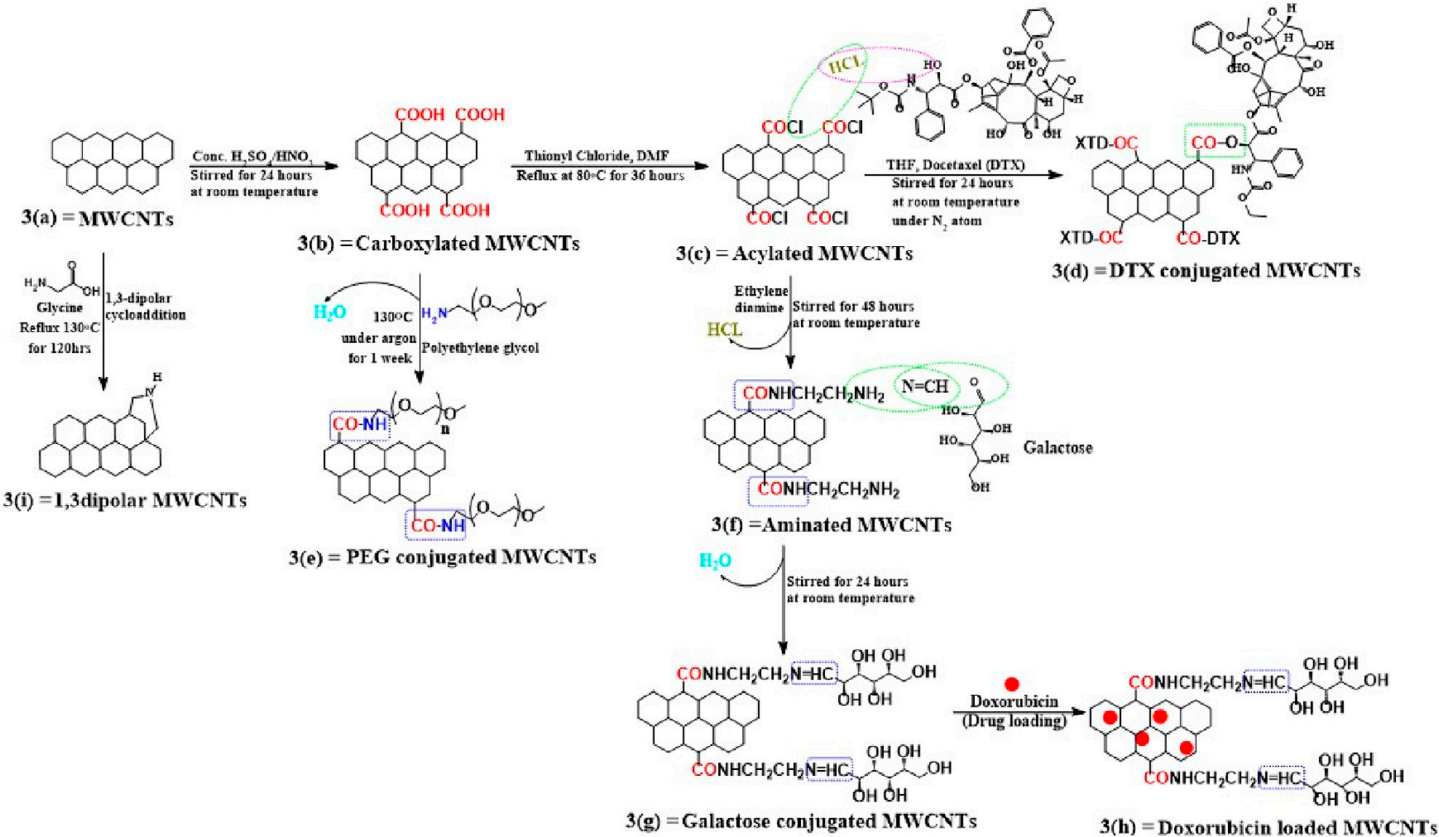
Mechanism for functionalizing pristine MWCNTs. Using this scheme, we show the different ways of covalently functionalizing (a) pristine MWCNTs and (b) describe how pristine MWCNTs are oxidized by strong acidic solvents and how carboxyl functionalized MWCNTs have been further covalently modified, whether through conversion to acylated form (c) or direct conjugation with polymers, such as PEG (e). A variety of methods have been used to conjugate acylated MWCNTs, including conjugation with linkers (f) or ligands (g), followed by drug loading, as shown in figure (h), or for the direct conjugation of docetaxel with acylated MWCNTs. (d) A second method is shown in this (i) that explains the cycloaddition reaction using glycine and this also directly modifies pristine MWCNTs without carboxylation or acylation.

Activated MWCNTs are novel nanocarriers that can be modified by different types of surfactants (including but not limited to, phosphoric acid esters, sodium lauryl sulfate, carboxylic acid salts and alkylbenzene sulfonates) and biopolymers (e.g., peptide, chitosan, nucleic acid) (Table 1). The introduction of several functional groups on the surface can be used to disperse the carbon nanocomposite material (Sun et al., 2002; Thakur et al., 2022). These functionalized MWCNTs have good biocompatibility, decreased toxicity and the capacity to cross cell membranes, which is important for targeted drug distribution (Vardharajula et al., 2012; Rele et al., 2024). Chemical methods, such as exohedral functionalization (noncovalent and covalent) and endohedral functionalization, are used to functionalize MWCNTs (Figure 2) (Jeon et al., 2011; Pandurangappa et al., 2011; Thakur et al., 2022). Figure 3 illustrates the common methodologies used to functionalize MWCNTs, which includes the covalent carboxylation method, using oxidizing compounds, such as sulphuric acid and nitric acid (Hirsch et al., 2005). These carboxylic groups allow for further covalent and noncovalent chemical reactions of the ligand and the linker (Menezes et al., 2019).

**TABLE 1:** Cell cytotoxicity (IC_50_) formulations in cancer cells (A549, HeLa, and MCF-7 cells).

S.No.	Formulations	A589 (μg/mL)	HeLa (μg/mL)	MCF-7 (μg/mL)
1	MTX	7.0 ± 0.8	7.1 ± 0.8	6.9 ± 0.3
2	Dox	2.7 ± 0.3	2.4 ± 0.3	2.6 ± 0.2
3	PTX	8.2 ± 0.2	8.4 ± 0.9	8.7 ± 0.9
4	MWCNTs-FA-MTX	1.5 ± 0.3	1.2 ± 0.2	1.4 ± 0.3
5	MWCNTs-FA-Dox	1.4 ± 0.3	0.7 ± 0.2	0.9 ± 0.1
6	MWCNTs-FA-PTX	2.5 ± 0.2	2.5 ± 0.2	2.4 ± 0.2

##### 1.3.2.1 Exohedral functionalization

**FIGURE 4:**
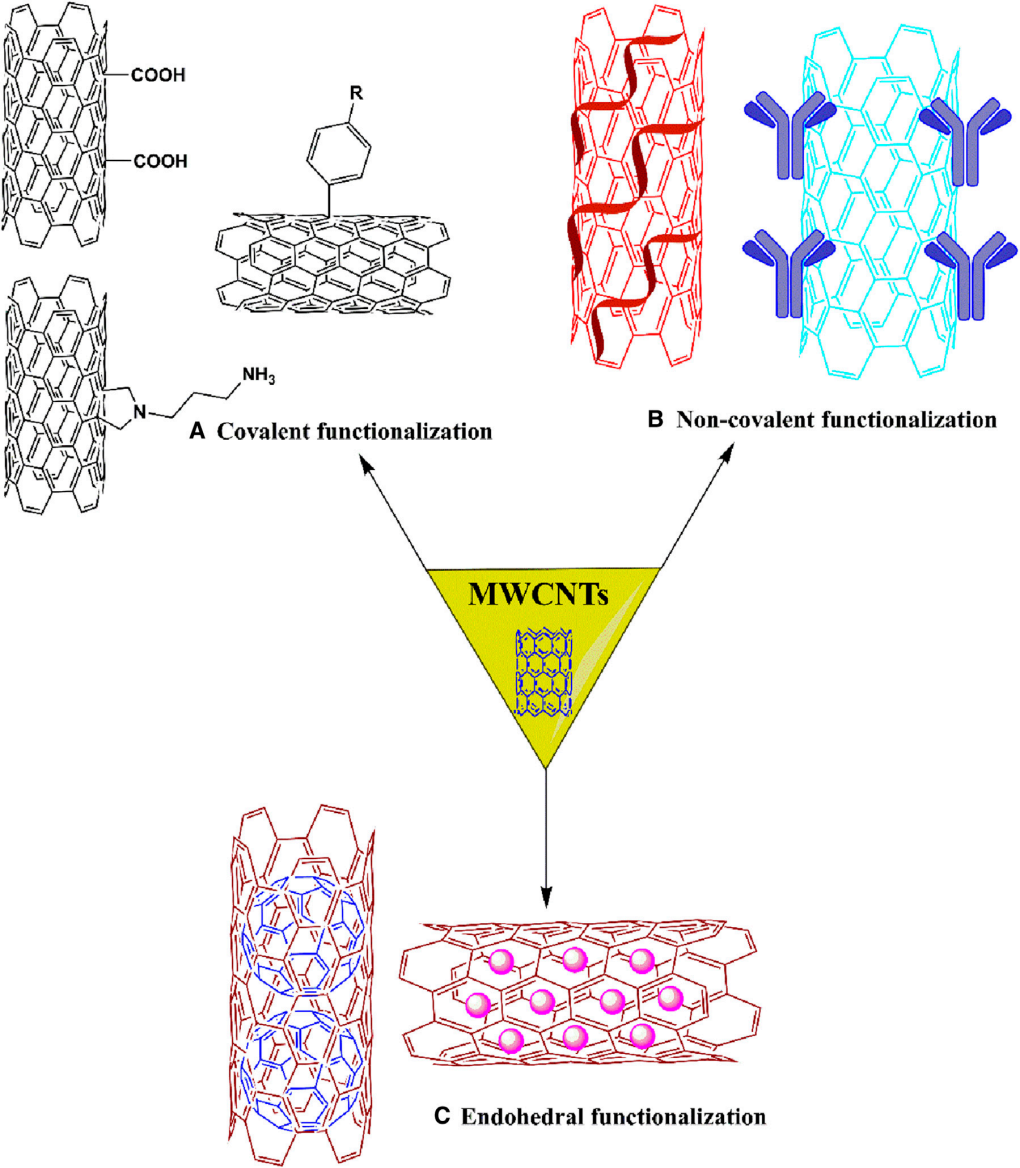
Schematic representation of exohedral [covalent **(A)** and non-covalent **(B)**] and endohedral **(C)** functionalization.

This process involves the grafting of molecules on the outer surface of the nanotubes with surfactants, using direct functionalization, covalent functionalization and non-covalent functionalization (Pandurangappa et al., 2011; Menezes et al., 2019). Exohedral functionalization can be classified according to the nature of interactions between the surface of MWCNTs and the functional groups or polymer chain, as shown in Figure 4. These interactions occur due to the formation of covalent or non-covalent bonds (Melchionna et al., 2013).

###### 1.3.2.1.1 Covalent functionalization

This method involves covalent interactions between the functional groups and the carbon skeleton of the nanotubes. The functional groups are formed by the incorporation of various chemical moieties or organic particles at different tips or in the sidewall of the MWCNTs. Covalent functionalization has been categorized as direct or indirect functionalization (Meng et al., 2009; Jeon et al., 2011). Direct sidewall covalent functionalization occurs by sp^2^ to sp^3^ hybridization and synchronization by removing the conjugated ligand (Sahoo et al., 2010). In contrast, indirect or direct covalent functionalization involves the chemical transformation of the open ends and sidewalls of holes, such as carboxylic groups, pentagon and hexagon graphene scaffolds, among others (Jeon et al., 2011).

###### 1.3.2.1.2 Noncovalent functionalization

Noncovalent modifications occur via supramolecular complexation formed by numerous noncovalent interactions, such as hydrogen bonds, π-π stacking interactions, vdW interactions and electrostatic interactions. Generally, surfactants, polymers and biopolymers are used for the dispersion of nanotubes, using self-assembling methods to produce hydrophobicity (Meng et al., 2009; Raza et al., 2016). The advantage of noncovalent functionalization is that it does not destroy the conjugated system of the MWCNTs sidewalls and thus, the structural properties of the final molecules are not significantly affected (Karousis et al., 2010). Furthermore, noncovalent functionalization is an alternative method for modulating the interfacial properties of the nanotubes (Meng et al., 2009).

##### 1.3.2.2 Endohedral functionalization

Nanotubes can be modified by bioactive molecules and nanoparticles that increase the hydrophilicity of the nanotubes, thus increasing their solubility in hydrophilic solvents. This process is illustrated in Figure 4 by the encapsulation of fullerene molecules and bioactive molecules inside MWCNTs (Delgado et al., 2008; Marega et al., 2014; Iglesias et al., 2019).

#### 1.3.3 The application of MWCNTs

At the beginning of the 21^st^ century, MWCNTs have been used by academic and industrial scientists to develop targeted drug delivery systems (Bianco et al., 2005; Bakry et al., 2007). Currently, the majority of MWCNTs - based research involves the formulation of drug delivery systems for various drugs and for gene therapy, diagnostic detection and drug vectors, among others (Hirlekar et al., 2009; Thakur et al., 2022). As a results of their significant chemical stability, large surface area and a high level of electronic aromatic structure, MWCNTs can be readily conjugated and interact with various ligands, enzymes, proteins, RNA and DNA, to delivery these molecules to the targeted sites (Lamberti et al., 2014).

## 2 MWCNTs as a conjugated ligand-based drug delivery system

A receptor is a biomacromolecule that recognizes and binds to signaling molecules and drugs (i.e., ligands) to produce a response that alters cellular physiology. For the targeted delivery of drugs, the drug-receptor interaction plays a critical role in mediating the efficacy of a drug. Consequently, there are numerous drugs that can be delivered to cells in a tissue and interact with a specific receptor to produce a specific response (Allen, 2002; Mehra et al., 2013).

**FIGURE 5:**
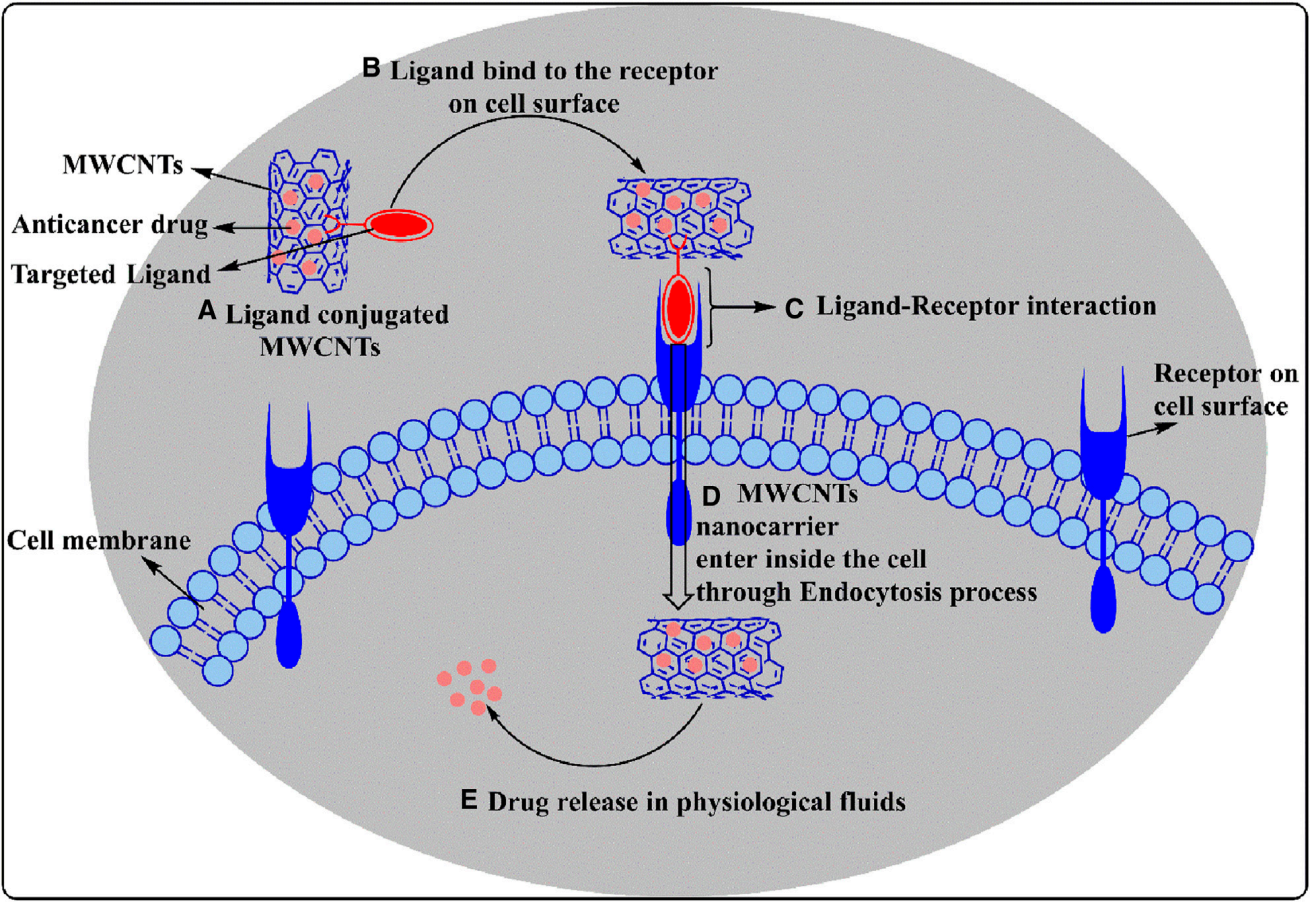
A diagrammatic representation of ligand conjugated MWCNT endocytosis mechanisms shows how drug-loaded ligand modified MWCNTs interact with receptors and enter the cells, and under which conditions the drug is released.

**FIGURE 6:**
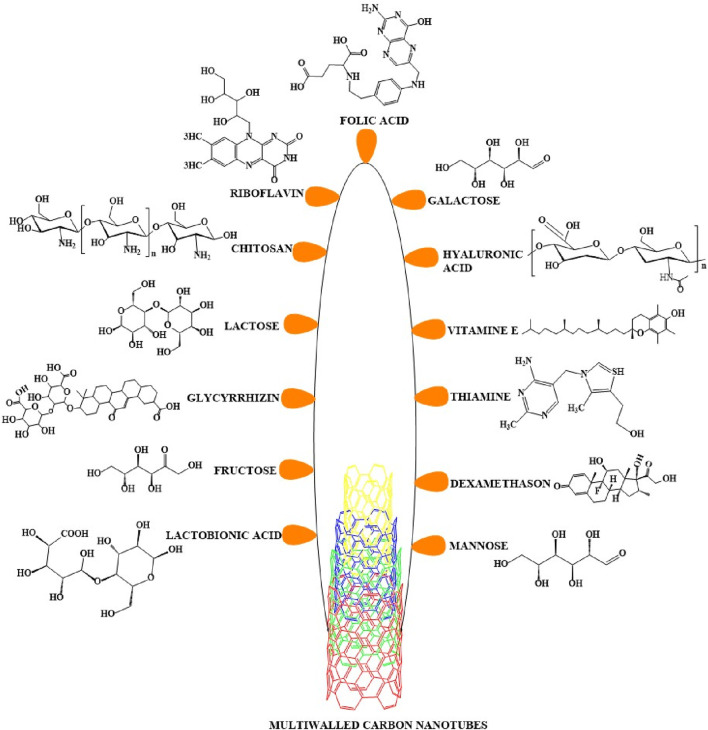
Multiwalled carbon nanotubes conjugated with various ligands. A variety of ligands can be covalently or noncovalently conjugated with MWCNTs to increase their recognition and binding to cellular receptors. The figure here includes all types of ligands that have been conjugated with MWCNTs for use in the treatment of cancer.

Target - based drug delivery system conjugate drugs and ligands with MWCNTs to deliver the drugs to the target. The internalization of the MWCNTs nanocarrier by the cells can occur by endocytosis, a process that involves the engulfment of the material by the cell membrane where it forms an endosome (Yaron et al., 2011; Maruyama et al., 2015) (see Figure 5). Once internalized, the conjugated MWCNT undergo biodegradation and release the drugs (Lamberti et al., 2014). This drug delivery method was first used to conjugate antibiotic and anticancer compounds with MWCNTs nanocarriers to treat certain types of microbial infections and cancers. Indeed, previous studies have reported that anticancer drugs, such as paclitaxel, docetaxel, cisplatin, doxorubicin, epirubicin, tamoxifen, methotrexate and quercetin, could be conjugated or loaded with the modified MWCNTs and their efficacy was validated by *in vitro* and *in vivo* experiments (Mehra et al., 2014; Raza et al., 2016). In order to deliver the drugs efficiently, various types of ligands, including but not limited to, folic acid, mannose, galactose and polymers, have been conjugated with MWCNTs (Figure 6) (Singh et al., 2016; Thakur et al., 2022).

### 2.1 Vitamins conjugated with MWCNTs

A variety of vitamins can be delivered to a specific target by conjugation with MWCNTs (Gao et al., 2018). In this section, we will discuss the targeted delivery of different vitamins-MWCNTs.

#### 2.1.1 Folic Acid

Numerous studies have reported that folic acid receptors are overexpressed in lung tumors, ovarian carcinomas, breast carcinomas, meningiomas, ependymal encephalon tumors, osteosarcomas, non-Hodgkin’s lymphomas, colon tumors, renal carcinomas and uterine sarcomas (Veetil et al., 2009; Fraczyk et al., 2017). Consequently, this led to the development of MWCNTs conjugated with folate that also contained anticancer drugs, thereby increasing the selectivity of drug delivery to the types of cancer.

**FIGURE 7:**
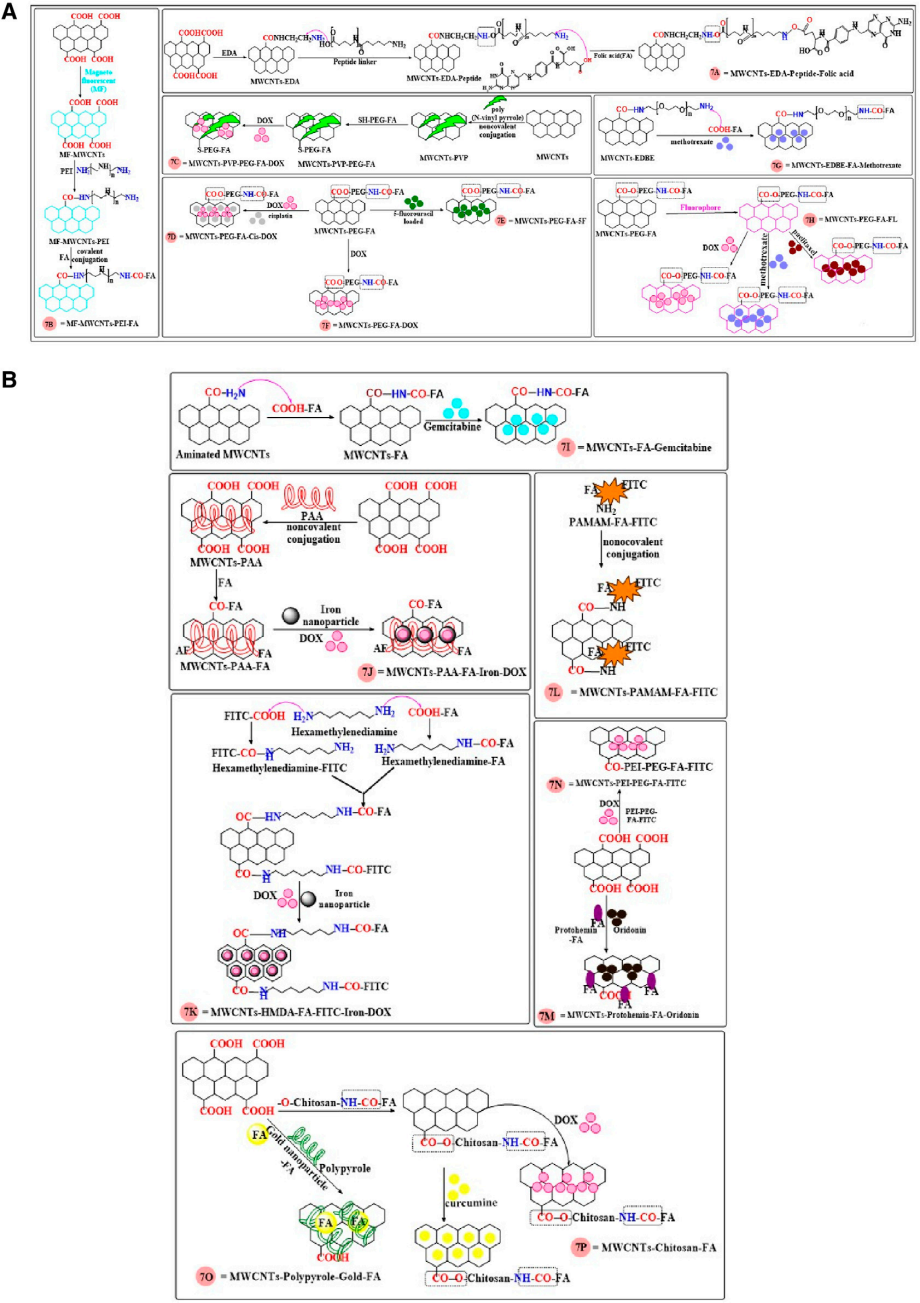
**(A, B)** The synthesis of MWCNTs conjugated with folic acid.

Recently, Fraczyk et al. (Fraczyk et al., 2017) formulated oxidized MWCNTs, using ethylenediamine in the presence of 4-(4,6-dimethoxy-1,3,5 triazin-2-yl)-4-methyl morpholinium tetrafluoroborate (DMTMM), to link a hydrophilic peptide linker (6-aminohexanoic acid and derivative of β-alanine) that was subsequently conjugated with folic acid (using a folate targeted ligand conjugation mechanism, as shown in Figure 7A. These nanomolecules had proteolytic efficacy in HT-29 colon cancer cells for at least 7 days. Furthermore, the conjugated MWCNTs did not significantly alter the viability of normal colonocyte cells, CCD 841 CoN. The metabolic stability of the nano-molecules was determined under physiological condition (37°C, pH.7.4, in PBS) in human heparinized plasma 7 days later. The results indicated that functionalized MWCNTs were stable and resistant to proteolytic degradation. Furthermore, the cellular uptake of folic acid functionalized MWCNTs was determined in Saos-2 cells (human osteosarcoma cell line), by measuring the fluorescence of fluorescein isothiocyanate (FITC), using a fluorescence microscope. The results indicated a high level of the functionalized folic acid inside the Saos-2 cells However, MWCNTs that were not functionalized with folic acid did not significantly increase the fluorescence intensity in the Saos-2 cells, compared to cells incubated with the folic acids functionalized MWCNTs. Zhang et al. (Zhang et al., 2018) reported the synthesis of multi-functionalized magneto-fluorescent - based carbon nanotubes (Figure 7A, B). In these molecules, amide bonds were formed between the free amino group of polyethyleneimine and the free carboxylic group of folic acid, yielding a folate-carbon nanotubes carrier for the delivery of doxorubicin cancer cells. Furthermore, the MWCNTs had the properties of magnetic resonance imaging/double modular fluorescence that could be used as a chemo-photothermal synergistic therapy that could decrease the growth of certain tumors. Wang et al. (Wang et al., 2017) reported the surface modification of MWCNTs, using poly (N-vinyl pyrrole) and conjugation with functionalized folic acid, using a thiolenated polyethylene glycol (Figure 7A, C), which increased the time in the circulation and the drug loading capacity. The loading ratio of doxorubicin was 453% and 365% of the drug loading ratio in MWCNTs coated with poly (N-vinyl pyrrole)-folic acid and poly (N-vinyl pyrrole), respectively. Moreover, in an *in vitro* drug release study in 10 mM of phosphate buffer at pH 5.5 and 7.4, the MWCNTs coated with poly (N-vinyl pyrrole)-folic acid released a significantly greater amount of doxorubicin at pH 5.5, compared to pH 7.4, indicating a pH responsive drug release that allows for the targeting of the acidic tumor microenvironment. The *in vitro* incubation of Hela cells with Dox - loaded MWCNTs formulation and pure MWCNTs formulation produced as 40% and 80% cell viability, respectively, results indicated pure MWCNTs formulations are nontoxic and better carrier for delivery of Dox. Yang et al. (Yang et al., 2017) reported the potential use of CNTs as a dual drug delivery system (DDDS), by incorporating cisplatin and doxorubicin (DOX) in MWCNTs, using a wet-chemical approach with folic acid and polyethylene glycol. DOX was attached to the external surface by non-covalent interactions, whereas cisplatin was encapsulated inside the nanotube. The antitumor efficacy of DDDS in MCF7 breast cancer cells was determined at pH 6.4 and 7.4. Based on the percent viable MCF7 cells, DOX produced significant antitumor efficacy at pH 6.5, compared to pH 7.4, after 72h of incubation. It has been reported that cisplatin can be encapsulated inside nano-vector MWCNTs, with external surface attachment of doxorubicin (Figure 7A, D). In addition, folic acid and polyethylene glycol were used to increase the drug loading capacity. The % DOX loading efficiency was increased in the folic acid modified MWCNTs, compared to non-modified MWCNTs was 192.67% and 174.07% of DOX loaded folic acid modified MWCNTs and DOX - loaded pristine MWCNTs, respectively. Furthermore, cisplatin was shown to be loaded in the pristine MWCNTs (92.80%) as compared to folic acid modified MWCNTs (84.56%). The cumulative release of the DOX-loaded folic acid MWCNTs and cisplatin - loaded folic acid MWCNTs was determined using a UV spectroscopy method at pH 7.4 and pH 6.5. At pH 6.5, the release of DOX and cisplatin was 22% and 26% respectively, which was significantly greater than that obtained at pH 7.4 (8% DOX release and 13% cisplatin release) after 72 h of incubation. The cytotoxicity experiments indicated that in MCF-7 cancer cells, the antitumor efficacy of DDDS was significantly greater at pH 6.5, compared to pH 7.4, as the acidic pH increases the release of DOX and cisplatin from the folic acid conjugated MWCNTs (Fraczyk et al., 2017).

5-Flurouracil (5-FU) was functionalized on the surface of MWCNTs, using folic acid and PEG for targeted delivery by Kaur et al. (Kaur et al., 2017). The sequestration of 5-FU in functionalized MWCNTs (using the carboxylic group of folates that were conjugated with the amino group of PEGylation bis - amine) (Figure 7A, E) was synthesized to target MCF-7 breast cancer cells. *In vitro*, 5-FU - loaded FA-PEG modified MWCNTs (20% cell viability) more inhibited the MCF-7 cells as compared to pure 5-FU (60% cell viability) at concentration 10 μg/mL. Following the i. v administration (3 mg/kg) of free 5-FU and 5-FU functionalized MWCNTs, in Wistar rats, the elimination half-lives (t_1/2_) were 5.62 and 16.48 h, respectively, These clearly indicated that the 5-FU loaded FA-PEG modified MWCNTs was more efficacious than free 5-FU in decreasing the proliferation of MCF7 cells. Mehra and Jain (Mehra et al., 2013) developed a novel, site effective delivery system by loading doxorubicin (DOX) PEGylated MWCNTs conjugated with folic acid (Figure 7A, F). The IC_50_ of DOX functionalized MWCNTs was 15 μg/mL, which was significantly lower than that for free DOX (50 μg/mL), due to its targeted interaction with the folic acid receptors on the cancer cells. The bio-distribution profile in mice indicated the accumulation of DOX in the liver, tumors and kidneys, after the i. v. administration of 5 mg/kg of the DOX functionalized MWCNTs. The elimination half-lives for the DOX functionalized MWCNTs and free DOX formulations were 14.96 h and 1.89 h, respectively. Thus, compare to free Dox, the levels of the DOX functionalized MWNCTs were greater in the tumors and its clearance time was greater than that for free DOX. The novel ethylenedioxy)-bis-ethyl ammonium (EDBE) modified MWCNTs (Figure 7A, G) were synthesized, using theranostic prodrug conjugation with methotrexate, folic acid (the target ligand), a radiotracer (Technitium-99m) and a fluorochrome (Alexa-fluor, AF488/647) (Das et al., 2013a). The *in vitro* stability indicated that the non-modified MWCNTs compounds had a stability of 91%–94% after 24 h of incubation in PBS at pH 7.4. The folic acid conjugated MWCNTs accumulated in MCF-7 and A549 cancer cells, as these cancer cells overexpress folic acid receptors (Kaittanis et al., 2009; Santra et al., 2009). In contrast, in Neuro 2A cells, which do not express folic acid receptors, there was no accumulation of the folic acid conjugated MWCNTs. *In vitro,* methotrexate and FA-modified MWCNTs-methotrexate inhibited the proliferation of MCF-7 cells (IC_50_ values of 7.36 and 1.95 µM, respectively) and A549 cell (7.26 and 2.13 µM, respectively). Thus, *in vitro*, the modified MWCNTs were more efficacious than free methotrexate in inhibiting the proliferation of MCF7 and A549 cancer cells. Das et al. (2013a) reported that 24 h after the i. v. administration of 5 mg/kg of free methotrexate and MWCNTs-conjugated methotrexate in 7, 12- dimethylbenzα anthracene (DMBA) carcinogen induced female Sprague-Dawley rats, the tumor growth inhibition of free methotrexate and MWCNTs-conjugated methotrexate was 25.37% and 38.37% after 24 h respectively. The inhibition of tumor growth, 1 week after 2 i. v. treatments with free methotrexate and MWCNTs-conjugated methotrexate was 80% and 50%. Thus, the MWCNTs-conjugated methotrexate formulation was significantly more efficacious than free methotrexate. Twenty-four hours after the i. v. administration of free methotrexate high levels was present in the stomach, intestine and heart. In contrast, the amount of methotrexate loaded MWNCTs in the stomach, intestine and heart was 0.04 ± 0.01, 0.07 ± 0.01, 0.05% ± 0.01% injected dose (ID)/g, respectively. According to these finding MWCNTs-conjugated methotrexate formulation are more effective in tumor and less accumulated in organs as compared to free methotrexate (Das et al., 2013a; Das et al., 2013b).

Das et al. (2013b) designed a bioactive functionalized MWCNTs (see Figure 7A, H) linked to estradiol (EA), folic acid (FA), hyaluronic acid (HA), a modified polymer (polyethyleneglycol), fluorophores (AF-647), and the anticancer drugs, methotrexate (MTX), doxorubicin (Dox) and paclitaxel (PTX). These multifunctionalized MWCNTs formulations were individually loaded with MTX, Dox or PTX. The % drug loading efficacy of PTX (91.4% ± 2.6%) was less than that for MWCNTs-FA-MTX (97.8% ± 2.1%) and MWCNTs-FA-Dox (97.8% ± 2.4%). The IC_50_ value of compounds in A549 cells, HeLa cells and MCF-7 cells is shown in Table 1, Based on the IC_50_ values, the MWCNTs-FA loaded with anticancer drugs were more efficacious than the pure anticancer drug alone.

The conjugation of folic acid to MWCNTs containing gemcitabine (Figure 7B, I), was developed to target human breast cancer cells (MCF-7) (Singh et al., 2013). The % drug loading efficiency of gemcitabine was greater in the modified MWCNTs (79.60 ± 0.45), compared to the non-modified MWCNTs (71.60 ± 0.25). *In vitro*, the incubation of MCF-7 cells with 80 μg/mL of MWNCTs loaded with gemcitabine produced a 40% decrease in cell viability, compared to 20% for free gemcitabine. Bio-distribution experiments in Sprague-Dawley rats indicated that 1 h after the i. v. administration of 1 mg/kg of gemcitabine formulated MWCNTs, the levels of gemcitabine were significantly lower in the liver, speen, stomach, kidney, compared to 1 mg/kg i. v. of pure gemcitabine. *In vivo* pharmacokinetic data indicated that the bioavailability and plasma levels of the MWCNTs formulated with gemcitabine was significantly greater than that of pure gemcitabine. Finally, the incubation of human red blood cells with conc. 500 μL of effect of MWCNTs formulated with gemcitabine or pure gemcitabine produced a level of hemolysis of 8.23% ± 0.65% and 17.34% ± 0.56%, respectively, indicating the gemcitabine formulated MWNCTs were less toxic than pure gemcitabine.

Lu et al. (Lu et al., 2012) designed MWCNTs noncovalently grafted with poly (acrylic acid), iron-oxide magnetic nanovectors and a folate ligand (Figure 7A, J), and the nanotubes were loaded with doxorubicin for targeted drug delivery. The drug loading capacity of the formulation was increased, as was the cytotoxic efficacy. The loading capacity of the MWCNTs for Dox was 96% when the Dox concentration was 0.5 mg, whereas the loading capacity for Dox was 92%, when the Dox concentration was 1 mg. The percentage cumulative release of Dox at pH 5.3 and 7.4, after 192 h of incubation, was 71% and 14%, respectively. The proliferation of U87 glioblastoma cells was inhibited by pure Dox (IC_50_ = 50 μg/mL) and Dox-loaded MWCNTs (IC_50_ = 15 μg/mL). The conjugation of glycine to MWCNTs/C_60_ fullerene, using the 1,3-dipolar cycloaddition method, was used to increase the targeted delivery of methotrexate (MTX) (Joshi et al., 2017). The MTX loading efficiency was greater in the C_60_ fullerene (79.98% ± 3.21%) formulation, as compared to the MWCNT formulation (75.47% ± 1.79%). The *in vitro* incubation of MDA-MB-231 breast cancer cells with the C60 fullerene-MTX compound produced a 2.6-folds greater decrease in cell viability, compared to pure MTX. The i. v administration of 2.5 mg/kg of MTX equivalent (either as pure MTX, C60 fullerene and MWCNTs formulation) to Wistar rats indicated that the C60 fullerene and MWCNTs formulations significantly increased the half-life (6.84 and 8.85 h, respectively) of MTX, compared to the pure MTX formulation (2.49 h). Furthermore, the clearance of MTX was significantly lower for the C60 fullerene and MWCNTs formulations (0.0047 mL/min and 0.0034 mL/min, respectively), compared to pure MTX (0.019 mL/min). The AUC for the C60 fullerene and MWCNTs formulations was greater (129.08 μg/mL/h and 179.92 μg/mL/h, respectively), compared to pure MTX (30.96 μg/mL/h).

Li et al. (Li et al., 2011) reported the synthesis of MWCNTs conjugated with folate and iron nanoparticles for the dual-targeted delivery of doxorubicin to HeLa cells. Initially, the folic acid and FITC were individually conjugated with hexamethylene diamine, then bonded with the carboxylic acid group of MWCNTs and encapsulated with iron nanoparticles or Dox (Figure 7B, K). The loading efficiency of Dox in the Dox - loaded MWCNTs nanocarrier and Dox loaded iron nanocarrier was 32 μg/mg and 24 μg/mg, respectively. The % cell viability of pure Dox and the Dox-loaded MWNCTs was 80% and 20% at 40 μg/mL concentration, results finding indicated Dox loaded MWCNTs are more effective in Hela cell as compared to pure Dox. MWCNTs-folic acid modified dendrimers (PAMAM) were synthesized to target KB-HFAR and KB-LFAR human mouth epidermal cancer cells (Shi et al., 2009). The aminated dendrimers (De) were noncovalently bonded to folic acid (FA) and FITC and subsequently linked with carboxylated MWCNTs (Figure 7B, L). The FA/FI/De modified MWCNTs compound produced a concentration-dependent inhibition of the proliferation of KB-HFAR cells due to the presence of the folic acid ligand that interacts with folic acid receptors. However, the FI/De modified MWCNTs nanocarriers did not interact with KB-HFAR cells as this formulation lacked a folic acid ligand. KB-LFAR cells did not interact significantly with either FA/FI/De or FI/De modified MWCNTs nanocarriers due to absence of folic acid receptor on KB-LFAR cancer cells. The proliferation the KB-HFAR and KB-LFAR cells was not significantly altered by incubation with FA/FI/De or FI/De modified MWCNTs nanocarriers (1–100 μg/mL). Overall, these data suggest that the MWCNTs formulation in this study were biocompatible and safe for targeted drug delivery. Wang et al. (Wang et al., 2019) designed a novel MWCNTs-folic acid-protohemin modified/encapsulated compound that was covalently conjugated with oridonin (Figure 7B, M), an anticancer drug, to target its delivery to human HepG2 cancer cells. *In vitro*, 12 μg/mL of oridonin loaded liposome or the folic acid modified oridonin MWCNTs formulation inhibited the proliferation of HepG2 cells by 42.3% ± 2.9% and 95.4% ± 5.9%, respectively. The folic acid modified MWCNTs had the highest uptake into HepG2 cells due to the presence of folic acid receptors on their surface. In BALB/c male mice bearing HepG2 cells, the i. v. administration of 0.01 g/kg/day of either oridonin loaded liposome or folic acid modified oridonin MWCNTs for 10 days decreased tumor growth by 32.5% and 90.0%, respectively.

A multifunctional drug delivery nanocarrier of doxorubicin - loaded MWCNTs, with covalently adhered polyethyleneimine, FITC, polyethylene glycol and folic acid (Figure 7B, N), was synthesized to target Hela cancer cells. *In vitro*, the proliferation of HeLa cells was inhibited by pure Dox (IC_50_ = 3.45 mg/L) and Dox - loaded folate conjugated MWCNTs (3.53 mg/L). In HeLa-HFAR (high folic acid receptors) and HeLa-LFAR (low folic acid receptor) cells, incubation with the Dox - loaded folate conjugated MWCNTs inhibited their proliferation by 84.5% and 54.2%, respectively. These results indicate that a low level of folic acid receptors decreases the efficacy of the MWCNTs conjugated with folic acid. An intravenous injection of 5 mg/kg equivalent of Dox, Dox + folate conjugated MWNCTs or MWNCTs alone produced a 42.5, 73.3% and 9.1% increase, respectively in the apoptosis of tumors cells in male BALB/c nude mice xenografted with Hela tumors. Twelve hours after the i. v. administration of 5 mg/kg of Dox-loaded folate conjugated MWCNTs in male BALB/c nude mice, Dox was present in the heart, kidney, liver and spleen, at 5.99, 1.50, 20.15% and 7.49%, respectively, of the injected dose per Gram of tissue (% ID/g) (Yan et al., 2018).

Docetaxel or coumarin-6 encapsulated with chitosan-folic acid modified MWCNTs, was reported to increase the delivery of docetaxel and coumarin-6 to A549 cancer cells, which overexpress folic acid receptor, thereby increasing the drug loading capacity. Chitosan links folic acid and MWCNTs via covalent bonds between the carboxylic group of the folates and the amino group of chitosan (forming an amide bond formation), whereas the hydroxy group of chitosan interacts with the carboxylic groups of MWCNTs, forming an ester bond (Figure 7B, O). The drug loading capacity of docetaxel and coumarin-6 were increased in functionalized MWCNTs (79.29% ± 1.3% and 83.62% ± 1.2%, respectively), compared to non-functionalized MWCNTs (59.72% ± 2.3% and 63.08% ± 3.2%, respectively). The proliferation of A549 cells was inhibited by docetaxel formulated in MWNCTs (IC_50_ = 0.56 μg/mL) and docetaxel (IC_50_ = 50.19 ± 2.5 μg/mL). The intracellular levels of docetaxel formulated in MWNCTs was increased in the A549 cells, due to the presence of chitosan and folic acid in the formulation (Singh et al., 2017).

Wang et al. (Wang et al., 2017) synthesized a compound where folic acid was noncovalently adhered to gold nanoparticles with polypyrrole that was grafted layer by layer with MWCNTs that contained doxorubicin bound by π-π stretching interactions, for cancer therapy (Figure 7B, P). *In vitro*, the release of docetaxel at pH 5.5 and 7.4 was 25% and 5%, respectively, after 72 h of incubation. This is important as the pH of cancer cells is typically acidic (Logozzi et al., 2018; Lyra et al., 2021) and thus, more drugs should be released at the tumor site. The *in vitro* cytotoxicity studies indicated that the docetaxel - loaded functionalized MWCNTs, at 50 μg/mL highest conc. used, inhibited the proliferation of Hela cells and H9C2 cardiomyoblast cells, by 70% and 65%, respectively. In contrast, the functionalized MWCNTs (maximal conc. 5 μg/mL–100 μg/mL) without docetaxel, decreased HeLa and H9C2 cardiomyoblast cell proliferation by 30% and 26%, respectively.

Ozgen et al. (Ozgen et al., 2020), synthesized a novel glycol-block copolymer conjugated MWCNTs for dual targeting delivery in breast cancer. For the preparation of glycol-block copolymer using poly (1-O-methacryloyl-b-D- fructo-pyranose-b-(2-methacryloxyethoxy)) benzaldehyde in which Dox was attached then these copolymer noncovalently coupled with MWCNTs further conjugated with folic acid for dual targeting of folate receptor as well as glucose transporter protein in MDAMB231 and MCF7 breast carcinoma cells. Developed copolymer and folic acid conjugated MWCNTs formulation showed higher dispersibility in aqueous medium as compared to pristine MWCNTs. Cell viability and Apoptosis studies were performed in MDAMB231 and MCF7 breast carcinoma cells and results were found as copolymer and folic acid conjugated MWCNTs formulation showed higher cell viability or apoptosis (96.58% in MDAMB231 or 87.46% in MCF7) as compared to pure Dox (72.80% in MDAMB231 or 42.68% in MCF7) & without folic acid conjugated copolymer MWCNTs formulation (81.24% in MDAMB231 or 85.00% in MCF7) after 24 h s of incubations, these finding data demonstrated developed formulations enhanced the dispersibility, apoptosis and provided dual targeted effect in breast cancer cells.

Zhou and coworker (Zhou et al., 2022), developed a novel nanocarrier functionalized carboxylated MWCNTs with polyethylene glycol, hyperbranched poly-L-lysine cross linked with adipic acid and finally conjugated with folic acid ligand, this functionalized MWCNTs nanocarrier loaded with Dox for targeted delivery of cancer cells like HepG2 (human liver cancer) and HEK293 (human embryonic kidney). Cell viability and Apoptosis studies were performed in HepG2 and HEK293 cancer cell and results was found as MWCNTs nanocarrier loaded with Dox showed higher cell viability (>20%) as compared to pure Dox (>40%) and pure MWCNTs nanocarrier showed less cytotoxicity in both the cell line (>90%). Prepared formulations are biocompatible and safe for delivery of anticancer drug.

#### 2.1.2 Riboflavin and thiamine

**FIGURE 8:**
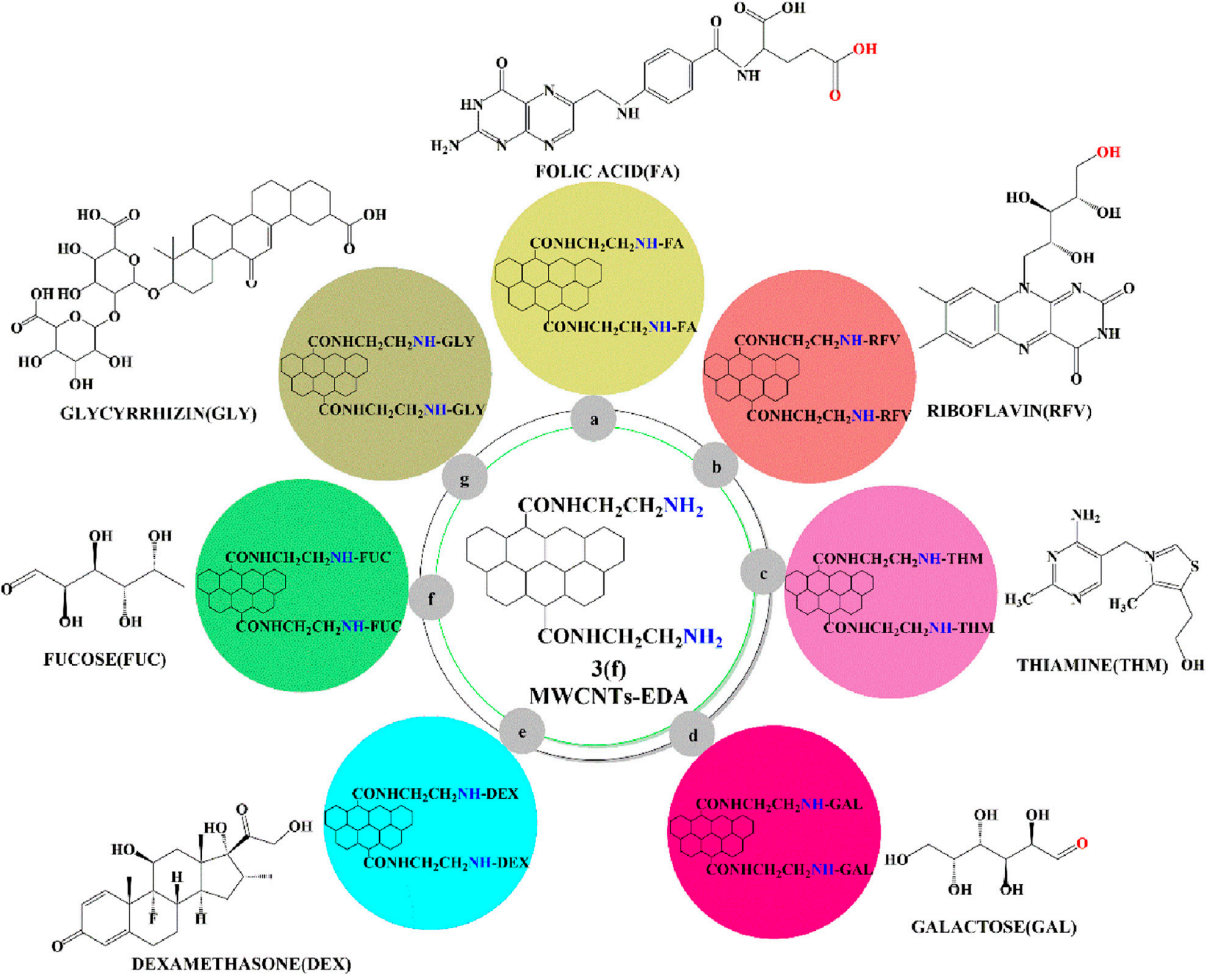
Ligands conjugated with an ethylenediamine linker containing MWCNTs.

Singh et al. (Singh et al., 2016 (a)) synthesized novel forms of the ligands, riboflavin (Figure 8B) and thiamine (Figure 8C), for the targeted delivery of paclitaxel to the breast cancer cell line, MCF-7. Paclitaxel - loaded MWCNTs were conjugated with the receptor targeted ligands, thiamine or riboflavin, and their levels were determined in the MCF-7 cells. In this formulation, the pristine MWCNTs were chemically modified by carboxylation, acylation and amination, and the ligand was ultimately conjugated with amine groups in the MWCNTs to form an amide bond, using an ethylenediamine linker. The *in vitro* incubation of MCF-7 cells with conc. (10–80 μg/mL) of pure paclitaxel or the MWNCTs-paclitaxel formulation decreased their viability by 40% or 20%, respectively. The MWNCTs-paclitaxel formulation was more efficacious than pure paclitaxel due to their nanosize, which increased their penetration across the tumor cell membranes, increasing the magnitude of tumor cell apoptosis. Following the i. v. administration of 10 mg/kg of pure paclitaxel to Sprague Dawley rats, the plasma conc. of paclitaxel was less than 5 μg/mL after 12 h, result indicated pure paclitaxel showed rapid clearance from blood plasma. In contrast, following the i. v. administration of 10 mg/kg i. v. of the paclitaxel loaded MWCNTs formulations, the plasma conc. of paclitaxel was 5 μg/mL at 36 h, showed the presence in blood for long duration as compared to pure paclitaxel. The % of human RBC hemolyzed after incubation of human RBC with conc. 0.5 mL of PTX functionalized in MWCNTs containing thiamine or 0.5 mL conc. of riboflavin, was 14.6 ± 0.84 and 11.17 ± 0.77, respectively, which was lower than that for 0.5 mL conc. of pure paclitaxel (20.49 ± 0.97) and 0.5 mL conc. of PTX functionalized in MWCNTs (37.39% ± 0.78%).

#### 2.1.3 D-α-tocopheryl polyethylene glycol 1000 succinate

Vitamin E (also known as D-α-tocopherol) conjugated with polyethylene glycol 1000 produced the compound, D-α-tocopheryl polyethylene glycol 1000 succinate (TPGS) (Pawar et al., 2016). It was a water-soluble non-ionic compound that increased the solubility, absorption, bioavailability and efficacy of camptothecin, taxanes, docetaxel, coumarin-6, paclitaxel, emodin, gambogic acid, doxorubicin, cisplatin, quercetin and toptecan. Furthermore, TPGS loaded with plumbagin/gambogic acid circumvents multidrug resistance by inhibiting P-glycoprotein in MCF-7 breast cancer cells (Dou et al., 2014; Singh et al., 2016).

**FIGURE 9:**
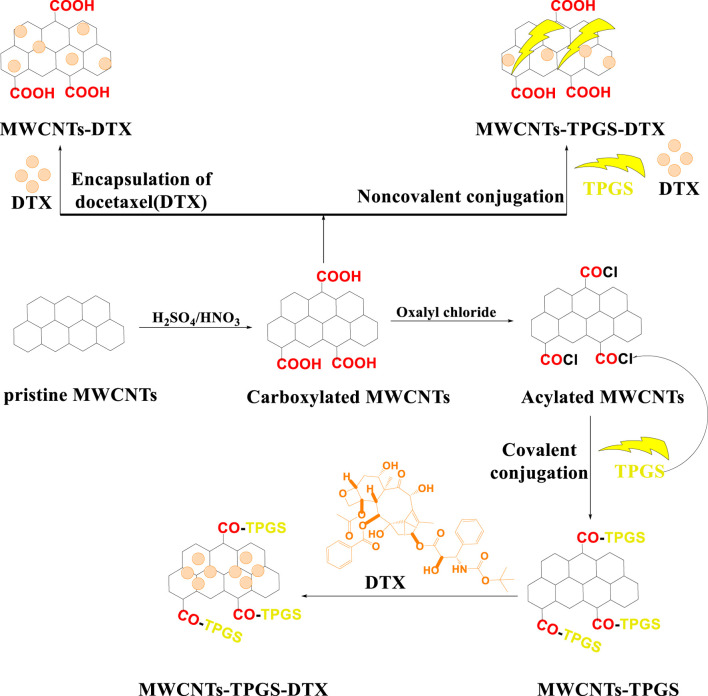
A schematic representation of MWCNTs covalently and noncovalently conjugated with the ligand, D-α-tocopheryl polyethylene glycol 1000 succinate (TPGS).

The covalent conjugation of TPGS on the noncovalent surface of the MWCNTs (Figure 9) was synthesized and loaded with docetaxel/coumarin 6 to increase the therapeutic efficacy of these drugs for A549 lung cancer cells (Singh et al., 2016 (b)). Cellular uptake assays indicated a higher concentration of the docetaxel-loaded TPGS-MWCNT compounds, compared to pure docetaxel, in the A549 cells. The loading efficiency of docetaxel in the TPGS coated MWCNTs and the non TPGS coated MWCNTs was 71.44% ± 1.8% and 76.53% ± 1.3%, respectively, whereas the loading efficiency of coumarin-6 in noncoated TPGS-MWCNTs was 62.61% ± 2.2% and 60.04% ± 2.8%, respectively. *In vitro*, A549 cells incubated with 5 μg/mL concentration of coumarin 6 loaded with TPGS coated MWCNTs, noncoated TPGS-MWCNTs and free coumarin 6. The results clearly indicated that a significantly greater amount of the MWCNTs loaded with coumarin 6 accumulated in the cytoplasm of the A549 cancer cells, compared to free coumarin 6. *In vitro,* pure docetaxel, docetaxel loaded TPGS coated MWCNTs and noncoated TPGS-MWCNTs inhibited the proliferation of A549 cells (IC_50_ values of 45.98 ± 1.7, 0.57 ± 0.02 and 1.49 ± 0.21 μg/mL, respectively). These results indicated that docetaxel - loaded TPGS coated MWCNTs were more efficacious than free docetaxel and noncoated TPGS-MWCNTs in inhibiting the proliferation of A549 cells.

Singhai et al. (2020) developed effective novel functionalized MWCNTs using α-tocopheryl succinate and chondroitin sulphate for improving therapeutic effect of Dox. Functionalized MWCNTs showed high cellular uptake in MDA-MB-231 breast cells as compared to pure Dox and which showed high apoptosis rate (53.40% ± 3.32%) compared to pure Dox (17.20% ± 1.22%) respectively. This functionalized MWCNTs has also enhanced the Dox loading capacity and provided pH dependent release, that is beneficial for cancer therapy.

### 2.2 Gonadotrophin releasing hormone (GnRH)

Studies have shown that the receptor for the protein, GnRH, is overexpressed in many types of cancers, particularly in the plasma membrane (Chen et al., 2002). Based on this, Moretti et al. covalently bonded GnRH to oxidized MWCNTs to target DU145 prostate cancer cells as they have been reported to overexpress GnRH receptors (Moretti et al., 2014). The GnRH - modified MWCNTs had a high magnitude of interaction with the cytoplasm of DU145 cells, compared to non-modified pristine MWCNTs. These results indicate that GnRH - modified MWCNTs can bind to the GnRH receptor and induce DU145 cell death. In addition, the GnRH - modified MWCNTs interacted with HeLa cells but not L929 cells, as these cells express low levels of GnRH. *In vitro*, the GnRH modified MWCNTs (0.010–0.050 mg/mL) decreased the proliferation of DU145 and HeLa cells by 85% and 35%, respectively, but it did not significantly alter the proliferation of L929 cells. The pristine MWCNTs (0.010–0.050 mg/mL) decreased the proliferation of HeLa, DU145 and L929 cells and GnRH (0.010–0.050 mg/mL) also decreased the proliferation of HeLa, DU145 and L929 cells, by above 80% cell viability in all cells respectively. The results of this study indicate that GnRH - modified MWCNTs have a high selectivity for detecting cancer cells expressing GnRH receptors.

### 2.3 Polymers

**FIGURE 10:**
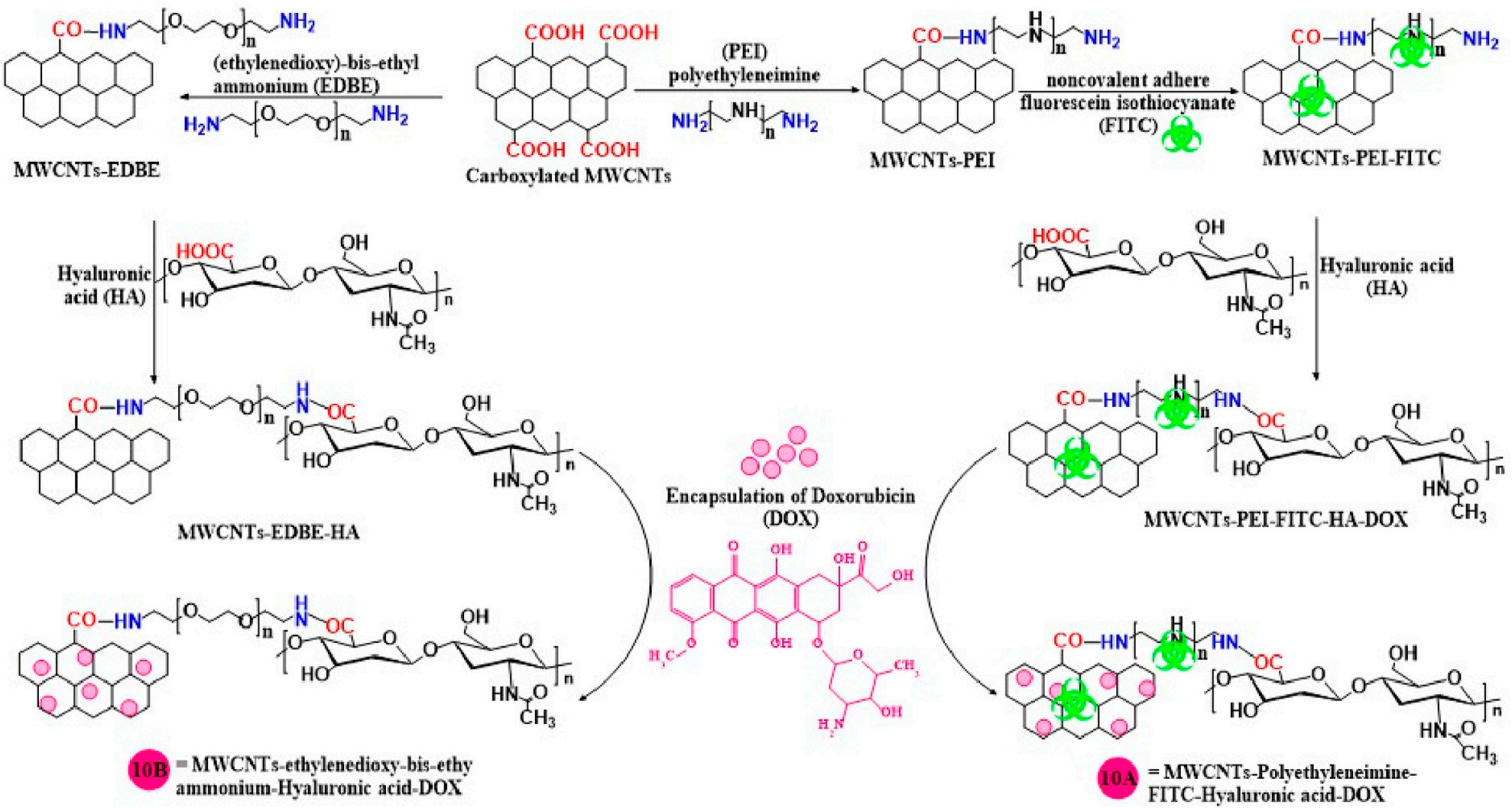
A schematic illustration of the synthesis of hyaluronic acid conjugated with MWCNTs.

Hyaluronic acid is a glycosaminoglycan polysaccharide that consists of glucuronic acid and N-acetylglucosamine (Smith et al., 2016). Hyaluronic acid can be used as a ligand to target the hyaluronic receptor in cancer cells and to increase the selectivity and therapeutic efficacy of the anticancer drug, doxorubicin (Dox) (Datir et al., 2012; Lokeshwar et al., 2014), because hyaluronic acid delivers drugs to targeted sites (Chadar et al., 2021). Datir et al. (Datir et al., 2012) covalently modified MWCNTs, using ethylenedioxy-bis-ethylammonium and then incorporated doxorubicin conjugated with hyaluronic acid, (Figure 10B), to target hyaluronan receptors in A549 lung cancer cells. In the lung cancer cells, the optimized MWCNT nanoformulation was 3.2-fold more efficacious in inhibiting the proliferation and 5-fold more efficacious in inducing apoptosis, compared to a solution of only doxorubicin.

Cao et al. (2015) developed an innovative nanocarrier for specific targeted drug delivery by conjugating hyaluronic acid and fluorescein isothiocyanate with modified MWCNTs using polyethyleneimine (10A). In this formulation, Dox was incorporated for targeted delivery to L929 (a mouse fibroblast cell) and HeLa cells which express CD44 receptors (Wang et al., 2016; Liu et al., 2018). The Dox loading capacity, using a UV spectroscopy method, was 72.0% in multifunctionalized MWCNTs. Dox release experiments indicated that a greater % of Dox was released at pH 5.8 (54.6%), compared to pH 7.4 (40.8%). This is important as the pH of the tumor microenvironment is typically acidic and thus, more Dox will be available to interact with the tumor cells. The cytotoxicity experiments indicated that the Dox - loaded multifunctionalized MWCNT and pure Dox decreased HeLa cell proliferation by 40% and 60%, respectively. The non-Dox multifunctionalized MWCNTs did not significantly alter the proliferation of the HeLa cells, suggesting that multifunctionalized MWCNTs should be safe and biocompatible. Using flow cytometry, it was shown that L929 cells expressed a low level of CD44, whereas HeLa cells expressed a high level of CD44. These results explain why multifunctionalized MWCNTs uptake in HeLa cell was seven times greater than that of L929 cells. Thus, the formulation reported in this study selectively targets cancer cells that overexpress CD44.

Singhai et al. (2020) synthesized MWCNTs using α-Tocopheryl succinate and hyaluronic acid, then loaded with Dox to develop a novel and effective nanocarrier for targeting the CD44 receptor which is overexpressed in MDA-MB-231 breast cancer. The cytotoxicity results indicated that functionalized MWCNTs showed high growth inhibition as compared to pure Dox with GI_50_ values of 0.810 ± 0.017 and 2.621 ± 0.153 μg/mL respectively. This finding revealed that hyaluronic acid is used to target the CD44 receptor for breast cancer therapy.

**FIGURE 11:**
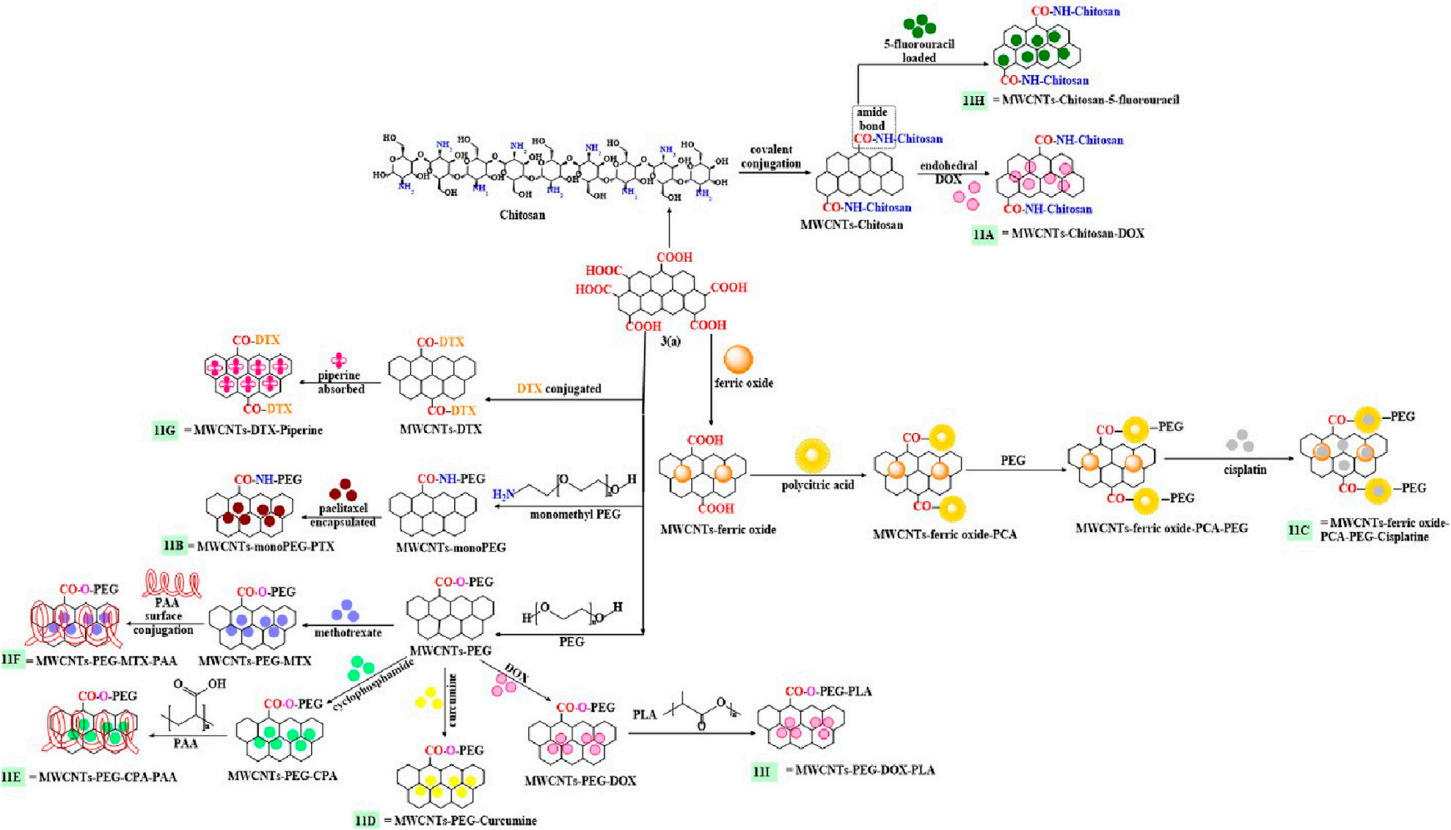
Chemically conjugation of carboxylated MWCNTs with different polymers.

Dong et al. (2017) formulated doxorubicin encapsulated with a chitosan modified MWCNTs (Figure 11A), where the acidic groups of the carboxylated MWCNTs were covalently conjugated with the free amino group of chitosan to form an amide bridge, and doxorubicin was encapsulated inside these modified nanotubes. *In vitro*, the percent loading capacity for MWCNTs: Dox, at ratios of 1:1, 2:1 and 1:3 were 24%, 33%, and 50%, respectively. The release of Dox from the MWCNT chitosan formulation was significantly greater at pH 5.8 (more than 75% Dox release) compared to pH 7.4 (less than 20% Dox release) after 72h of incubation. The uptake of pure Dox was higher than that of Dox - loaded chitosan MWCNTs, after 4 h of incubation with BEL-7402 cancer cells. However, the uptake of the Dox - loaded chitosan MWCNTs by BEL-7402 cells was greater than that of pure Dox, most likely due to the prolonged release of Dox from the nanocarrier system. Pure Dox, chitosan modified MWCNT and Dox - loaded chitosan MWCNTs produced a 10%, 75% and 9% decrease, respectively, in the proliferation of BEL-7402 cancer cells. PEG containing MWCNTs were synthesized by Lay et al., (2010) for the targeted delivery of paclitaxel to MCF-7 and HeLa cancer cells, using monomethyl polyethylene glycol (PEG) grafted MWCNTs (Figure 11B) formed by an endohedral process. The percentage of paclitaxel loaded by the PEG modified MWCNTs was 45% and the release of paclitaxel from this formulation at pH 5.0, 7.0 was 42% and 38%, respectively, compared to only 11% for paclitaxel as free paclitaxel had a lower aqueous solubility compared to paclitaxel loaded in the MWCNTs and the slower rate of release free paclitaxel. However, the PEG - modified MWCNTs provided a sustained release of paclitaxel over a period of 40 days at pH 5 and 7. Pure paclitaxel inhibited the proliferation of MCF7 and HeLa cells (IC_50_ values of 0.10 μg/mL and 0.021 μg/mL respectively), as did the paclitaxel loaded MWCNTs in MCF7 (IC_50_ value of 0.080 μg/mL) and HeLa cells (IC_50_ value of 0.010 μg/mL).

A multifunctionalized magnetically modified nanocarrier for targeted drug delivery to cancer cells was reported by Rezaei et al., (2018) Pristine side - walled MWCNTs were modified with cis-Pt (1,7-phenanthroline), ferric oxide, PEG, and poly (citric acid), followed by encapsulation of cisplatin (Figure 11C). The cisplatin loading capacity of the multifunctionalized MWCNTs was 86% and 76.6% of the cisplatin was released at pH 5.6, compared to 16.5% at pH 7.4, for up to 50 h. *In vitro*, the multifunctionalized MWCNTs loaded with cis-platin (concentration - 25, 50, 100 μg mL^−1^), inhibited the proliferation of MDA-MB-231 and HeLa cancer cells (less than 20% viability for these cell lines). In contrast, non-drug loaded multifunctionalized MWCNTs decreased the viability of MDA-MB-231 and HeLa cells 4% and 2%, respectively Thus, *in vitro,* the carrier alone was nontoxic, suggesting that it would be safe carrier for drug delivery.

The compound, curcumin (which has anticancer efficacy; (Allegra et al., 2017; Hindumathi et al., 2018; Rodrigue et al., 2019)) was incorporated into a biocompatible polyethylene glycol functionalized MWCNTs (Figure 11D) nanocarrier to target C6 cancer brain cells (Hindumathi et al., 2018). This formulation, compared to curcumin alone: 1) increased the drug loading of curcumin by 30%; 2) increased the uptake in the C6 brain cancer cells and 3) increased the solubility of curcumin in the aqueous medium. However, curcumin alone was not present in the C6 brain cancer cells as it did not cross cell membrane. The curcumin loaded MWNCTs, at 50 μg/mL, produced as 95% inhibition of the proliferation of C6 brain cancer.

The covalent conjugation of a poly (acrylic acid)-PEG copolymer with MWCNTs was synthesized to increase the delivery and efficacy of cyclophosphamide (Figure 11E) and methotrexate (Figure 11F) (Azghandi et al., 2017). The acylated MWCNTs reacted with the carboxylic acid moieties of PEG to form an ester link grafted with polymer polyacrylic acid to form a novel polymeric nanocarrier. The drugs, cyclophosphamide and methotrexate were loaded on to the MWCNTs and their % release in the buffer media at pH 4, was >80% for cyclophosphamide and >80% for methotrexate, after 1 h of incubation and at pH 7.4, 80% of both drugs (cyclophosphamide and methotrexate) was released after 3 h of incubation in PBS. Both drugs were released in a sustained pattern at pH 4 and their release was faster at pH 7.4.

Raza et al. (2016) designed a carboxylated MWCNTs containing piperine and covalent side walls modified with docetaxel (Figure 11G) for the targeted delivery of docetaxel in MCF-7 and MDA-MB-231 triple-negative breast cancer cells. *In vitro* experiments indicated that pure docetaxel, piperine and modified MWCNTs inhibited the proliferation of MCF-7 (IC_50_ values of 25, 121 and 8 μg/mL, respectively) and MDA-MB-231 (IC_50_ values of 15, 72.6 and 6 μg/mL, respectively) cells. The incubation of rat red blood cells with 2% (w/v) of the docetaxel conjugated piperine - modified MWCNTs for 1 h at 37°C, lysed <5% of the cells. *In vivo* pharmacokinetic experiments in male Wistar rats indicated that following the i. v. administration of 5 mg/kg equivalent of docetaxel (pure docetaxel and the Dox MWCNT formulation), there was a 50% decrease in the clearance and a 6.4-fold greater AUC for the Dox MWCNT formulation, compared to pure docetaxel.

Polymeric nanocarriers, built on chitosan covalently attached to MWCNTs, were loaded with the anticancer drug, 5-fluorouracil (Figure 11H) (Nivethaa et al., 2016). The 5-fluorouracil loading efficiency was 97% for the chitosan modified MWCNTs. At pH 5.0, 71.2% of the loaded 5-fluorouracil was released after 72 h in the release medium. The proliferation of MCF7 cells was decreased by 50%, after incubation with 100 μg/mL) of the 5-fluorouracil - loaded formulation.

Pistone et al. (2016) developed a drug delivery carrier, where doxorubicin was encapsulated with PEG and polylactic acid (PLA) modified MWCNTs (Figure 11I), for the targeted release of doxorubicin to HepG2, SH-SY5Y and HT-29 cancer cells. The loading capacity of the Dox - loaded functionalized MWCNTs was 25.4 wt%. The incubation of HepG2, SH-SY5Y and HT-29 cancer cells with 1–10 μg/mL of the Dox - loaded MWCNTs or Dox alone, decreased cell proliferation by <20%. The modified MWCNTs without Dox did not significantly alter the proliferation of HepG2, SH-SY5Y and HT-29 cancer cells, compared to Dox - loaded MWCNTs and Dox alone. The results indicated that pure MWCNTs modified formulation are nontoxic and a safe carrier for the delivery of Dox.

Selim et al., (2023) was formulated biotinylated chitosan noncovalently functionalized with MWCNTs for breast carcinoma treatment with using neratinib as a model drug which is having capacity to inhibit the tyrosine kinase. Firstly, MWCNTs was modified to carboxylated MWCNTs then noncovalently attached with biotinylated chitosan and finally loaded neratinib. Then prepared formulation was characterized by different spectroscopy technology and determine drug loading efficiency was 95.6%. *Invitro* release results of developed formulations shown in acidic condition showed high release upto 90% drug release at 72 h s but in physiological condition drug release below 40% at 72 h s, this results clearly showed developed formulation provided pH depended on release or it is essential for cancer treatments. SkBr3 cell line (human breast cancer cell line) was used to perform the cell cytotoxicity of free neratinib and neratinib loaded formulations, finding results was observed that free neratinib (IC50 value - 548.43 mg/mL) showed low cytotoxicity as compared to neratinib loaded formulations (IC50 value - 319.55 mg/mL & 257.75 mg/mL) respectively. Biotinylated chitosan noncovalently functionalized with MWCNTs formulations showed higher cell cytotoxicity as compared to without biotinylated chitosan functionalized MWCNTs formulations. Finding results clearly indicated biotinylated chitosan functionalized MWCNTs formulations enhanced the cytotoxicity as well as provided pH depended on releases.

Lyra et al., (2021) were formulated novel and effective guanidinylated dendritic conjugated MWCNTs for cancer targeting and Dox was loaded into modified formulations. Guanidinylated dendritic conjugated MWCNTs formulations showed higher drug loading efficiency as 99.5% as compared to unmodified MWCNTs as 78.7% respectively. Guanidinylated dendritic conjugated MWCNTs formulations enhanced the dispersibility in aqueous medium compared to pure MWCNTs, it is also enhanced the apoptosis and cell viability compared than pristine MWCNTs, Guanidinylated dendritic conjugated MWCNTs formulations showed higher cell viability in PC3 and DU145 human prostate carcinoma cells upto 40% and in normal cells HEK293 showed negligible cytotoxicity more than 90%. Finding results clearly shown prepared formulation enhanced the cell cytotoxicity, dispersibility as well as loading capacity.

### 2.4 Carbohydrate

The carbohydrates, galactose, lactose, mannose and fucose, among others, can be conjugated or modified to MWCNTs to form glyconanotubes, to increase the cellular uptake and efficacy of certain anticancer drugs (Chen et al., 2013).

#### 2.4.1 Galactose

**FIGURE 12:**
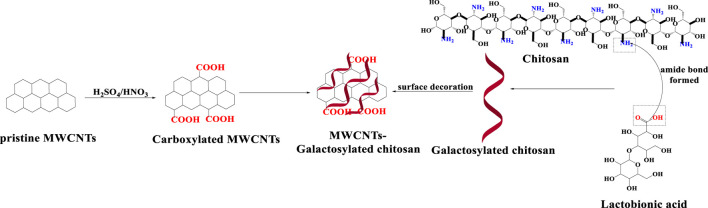
An illustration of MWCNTs decorated with galactosylated chitosan.

Qi et al. Qi et al. (2015) designed doxorubicin-loaded MWCNTs conjugated with galactosylated chitosan to target the liver cancer cell line (HepG2). Initially, the pristine MWCNTs undergo oxidation in the presence of sulphuric and nitric acid. Subsequently, they are non-covalently conjugated with galactosylated chitosan and finally, doxorubicin is loaded into this nanoformulation, as shown in Figure 12. The loading ratio of the Dox-loaded galactosylated MWCNTs was 25% ± 2%. The cumulative percentage release of Dox from the loaded galactosylated MWCNTs, at pH 7.4, 6.5 and 5.5 was 30%, 35% and 55%, respectively, after 24 h of incubation in a phosphate buffer saline media. % decrease in cell viability in HepG2 cells after incubation with Dox-loaded galactosylated MWCNTs (0.04–40.00 μg/mL), pure Dox (0.04–40.00 μg/mL) and pure galactosylated MWCNTs (0.04–40.00 μg/mL) was 9%, 10% and 80% respectively. These results indicate that pure galactosylated MWCNTs are nontoxic and effective carrier for delivery of anticancer drugs.

Jain et al. (2009), synthesized covalently modified MWCNTs galactose to increase the dispersibility of MWCNTs in aqueous medium. The carboxylated MWCNTs were converted to acylated MWCNTs by chemical modification, which formed covalent bonds with the amino group of ethylenediamine and the free amino group was conjugated with the aldehyde group of galactoses. Galactosylated MWCNTs (Figure 8D). The dispersion of pristine, acylated and carboxylated MWCNTs was determined using the visualization technique, The pristine MWCNTs and acylated MWCNTs were poorly dispersed in solutions at pH 4, 7 and 9. The carboxylated MWCNTs had a high degree of dispersion at pH 9 due to the greater ionization of the carboxylic acid residues. The aminated MWCNTs dispersion was greater at pH 4, compared to pH 7 and 9, because of the greater extent of protonation and ionization of the aminated groups at pH 4, compared to pH 7 and 9. The dispersion of the galactosylated MWCNTs was highest at pH 9, compared to pH 4 and 7, as the pKa value of galactose is approximately 12.35, which is similar to pH 9.

Thakur et al. (2022) was reported Carboxylated MWCNTs functionalized with lysine further conjugated with three different carbohydrate ligands such as galactose, mannose, lactose, and Dox used as model drug for targeting the MDAMB231 & MCF7 breast cancer cell lines. These functionalized MWCNTs were characterized with different spectroscopy techniques. Galactose, Mannose, Lactose functionalized MWCNTs showed higher drug loading capacity as compared to lysine functionalized MWCNTs as well as carboxylated MWCNTs as 96.78%, 97.29%, 95.56%, 93.30% and 90.83% respectively and these prepared formulations also provided pH depended on release. Dox loaded ligand (galactose & mannose) conjugated MWCNTs showed higher percentage of cell viability in both breast cancer cell line (MDAMB231 & MCF7) compared to pure Dox at higher concentration more than 12.5 μg/mL but lactose functionalized MWCNTs showed minimal cytotoxicity with pure Dox and without Dox loaded MWCNTs formulations showed very less cytotoxicity more than 90% in both breast cancer cells, It means pure formulations does not causes any cytotoxicity with cancer cells is safe, biocompatible and provided effective targeted delivery of drug to the targeted sites.

#### 2.4.2 Lactose

**FIGURE 13:**
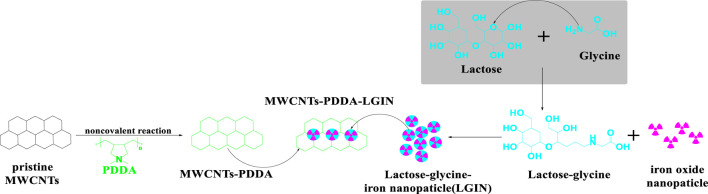
Conjugation sequences for the modification of MWCNTS-PDDA and lactose-glycine functionalized iron nanoparticles.

Multifunctional MWCNTs, combined with iron oxide - based superparamagnetic nanoparticles and lactose-glycine (Figure 13), coated with poly-diallyl-dimethyl-ammonium-chloride (PDDA), were developed by Liu et al. (2014). The % cell viability of HEK293 and Huh7 cells following incubation with 150 ug/mL of multifunctionalized MWCNTs was >25% and <90%, respectively. There was a significantly greater accumulation of the multifunctionalized MWCNTs in liver tumors in Harlan BALBc mice, compared to normal liver tissue (2.77-fold), after the i. v. administration of 10 mg/kg of multifunctionalized MWCNTs.

#### 2.4.3 Fucose

Gupta et al. (2014) synthesized a MWCNT with a fucose sugar moiety that incorporated sulfasalazine (Figure 8F), for targeted delivery to J774 liver cancer cells. The loading capacity of sulfasalazine into the fucosylated MWCNTs was 87.77% ± 0.11%. *In vitro*, 100 µM of fucosylated MWCNTs loaded with sulfasalazine produced a 25% decrease in the viability of J774 cells (a macrophage cell line isolated from humans). However, unloaded fucosylated MWCNTs and pure sulfasalazine, at 100 μM, only decreased J774 cell viability by 95% and 40%, respectively, indicating that the pure formulation is non-toxic and fucosylated MWCNTs loaded with sulfasalazine are more effective in J774 cells than pure sulfasalazine. The *in vivo* pharmacokinetic experiments indicated that after the i. v. administration of either 20 mg/kg of pure sulfasalazine, MWCNTs loaded sulfasalazine or fucosylated MWCNTs - loaded sulfasalazine, in male Sprague-Dawley rats, the 1) rates of clearance were 168.01 ± 0.93, 155.62 ± 0.61 and 124.80 ± 0.74 μg/mL, respectively; 2) half-lives were 5.06, 6.33, 9.92 h, respectively and 3) AUCs were 1004.55, 1262.47, 1646.99 μg h/mL, respectively. Overall, these data clearly indicated that the fucosylated MWCNTs - loaded sulfasalazine had a longer half-life, a greater AUC, and a lower clearance, compared to the other formulations.

### 2.5 Dexamethasone

Dexamethasone is a synthetic corticosteroid that is used in the treatment of numerous diseases, including certain types of solid and non-solid cancers (Ruy et al., 2003; Park et al., 2006). It was reported that dexamethasone can be conjugated on the surface of MWCNTs using an ethylenediamine linker, to form aminated MWCNTs, in the presence of 2-iminothiolane (Figure 8E). Doxorubicin (Dox) was incorporated into the functionalized MWCNTs using a nano extraction method, where doxorubicin was dissolved in acetone and triethylamine and added to functionalized MWCNTs and stirred for 24h at room temperature (Lodhi et al., 2013). *In vitro,* the incubation of A549 cells with 1–10 μg/mL of formulated Dox and pure Dox decreased their viability by 40% and 45%, respectively. The % hemolysis of human red blood cells by Dox alone (17.1% ± 0.2%) was significantly greater than that of the Dox - loaded dexamethasone-MWCNTs (9.5% ± 0.6%).

### 2.6 Glycyrrhizin

Glycyrrhizin is a compound present in the plant, *glycyrrhiza glabra,* and studies suggest that it has *in vitro* and *in vivo* anticancer efficacy (Wang et al., 2019; Thirugnanam et al., 2008; Cai et al., 2017). Chopdey et al. (2015) developed MWCNTs conjugated with glycyrrhizin and encapsulated with doxorubicin, for targeted delivery to HepG2 cancer cells. Glycyrrhizinated MWCNTs (Figure 8G) were developed by modifying the pristine MWCNTs to carboxylated MWCNTs, which were aminated with ethylenediamine and the N-terminal of the aminated MWCNTs was conjugated with carboxylic group of the glycyrrhizins to form amide bonds. The Dox loading efficacy was greater for Dox-Glycyrrhizinated MWCNTs (87.26 ± 0.57%), compared to Dox-MWCNTs (77.08 ± 0.62%). 1 mg/ml of Dox-Glycyrrhizinated MWCNTs (9.82 ± 0.67) produced a lower % hemolysis of human red blood cells than Dox-MWCNTs (18.36 ± 0.33%). Dox alone, Dox-MWCNTs and Dox-Glycyrrhizinated MWCNTs significantly decreased the proliferation of HepG2 cells (IC_50_ values of 4.19 ± 0.08, 4.15 ± 0.01 2.7 ± 0.03 µM, respectively).

## 3 Biological disposition and toxicity of MWCNTs in cancer drug delivery

MWCNTs have several benefits for use in cancer drug delivery (Panigrahi et al., 2020). These have been shown to have good biocompatibility and slow biodegradability and can undergo enzymatic fragmentation in the body over time, which can reduce the risk of long-term toxicity and make them a safer option for drug delivery use (Cao et al., 2019; Aoki et al., 2020). MWCNTs have the versatility of easy detection by a different imaging technique, which makes them useful for tracking their movement in the tumor tissue. Moreover, the excellent electrical, and thermal characteristics enable MWCNTs serve as cargos for cancer drugs and deliver them directly to tumor cells, and they can be used to heat up tumors during thermotherapy to kill cancer cells (Masoudi et al., 2023).

The use of MWCNTs as biomaterials has seen a significant rise due to their demonstrated efficacy as drug delivery systems and their biological safety. While it cannot be conclusively claimed that MWCNTs are completely safe biological systems, extensive studies have shown no evidence of biological risk, leading most researchers to deem them safe for use as long as the appropriate delivery method and site are used (Ciofani et al., 2010; Saito et al., 2014). Studies have shown that the toxicity of CNTs is related to their physicochemical properties, such as dimensions and functionalization, as well as their method of entry and site of injection (Shvedova et al., 2005; Liu et al., 2008; Chall et al., 2021). Studies have shown that the toxicity of MWCNTs is largely dependent on their length, diameter, and surface chemistry. For instance, longer MWCNTs have been found to be more toxic than shorter ones, while smaller diameter MWCNTs are more toxic than larger ones. When longer MWCNTs (20 μm) are injected, it causes more serious toxicity like aggregation because phages are not able to engulf the micro size fibers. 825 nm longer MWCNTs causes inflammation because microphages could not envelop more than 220 nm, while 50 nm diameter of MWCNTs showed cytotoxicity or inflammogenic due to high crystallin nature, but 150 nm–220 nm of MWCNTs showed less cytotoxicity and inflammogenic (Liu et al., 2013; Kobayashi et al., 2017; Ali, 2023). Moreover, the surface chemistry of MWCNTs can influence their toxicity as it affects their interactions with biological systems. Managing these properties can be a useful approach in minimizing the toxicity of MWCNTs (Yan et al., 2011; Bussy et al., 2012; Louro, 2018; Ali, 2023). Extensive biological studies and regulatory standards must be established to validate the safety of MWCNTs. Extreme caution is necessary when taking the risky step towards clinical application involving the entry of CNTs into the circulation to ensure safe use (Van et al., 2011).

The presence of metallic impurities together with hydrophobic accumulation contributes significantly to the toxicity of MWCNTs, which can be reduced by the functionalization. Several studies or being conducted to develop safe and effective CNT nanocarriers by purifying metallic impurities (Mehra et al., 2015; Francis et al., 2017). Functionalized CNTs are considered as promising nanocarrier for biomedical applications. Modifying the surface of MWCNTs with targeting ligands, biodegradable and biocompatible polymers can improve biocompatibility and reduce toxicity. In addition, the utilization of specific sizes and shapes of MWCNTs has been shown to improve biodistribution and decrease toxicity (Liu et al., 2013; Francis et al., 2017).

## 4 Distribution and elimination of MWCNTs

The subcutaneously injected MWCNTs into mice showed that MWCNTs did not accumulate in organs except for lymph nodes, and there was no observed injury. The study demonstrates that subcutaneous injection of MWCNTs induces short-term immunological reactions that can be eliminated over time and SC route of administration was believed to be safer than systemic administration (Meng et al., 2011). In a recent study, the biodistribution of chitosan-MWCNT, and chitosan crosslinked MWCNT loaded with anti-gastritis drug, HEP (hericium erinaceus polysaccharide) was studied in mice following intraperitoneal injection. Real-time fluorescence imaging results indicated that CS-MWCNT-HEP had higher accumulation in liver and spleen. Authors claimed that the accumulation could enhance the body’s immunity and metabolism, but it is not clear how the emptied MWCNT will be cleared from the body (Ren et al., 2020).

Systemic administration via intravenous injection and distribution in the bloodstream has the potential to accumulate in various organs and tissues. The exact distribution pattern may depend on factors such as the size (length and diameter), shape, surface properties, and functionalization of the MWCNTs, as well as the dose and injection site (Jacobsen et al., 2017). Studies have shown that after injection, MWCNTs can accumulate in organs such as the liver, spleen, lungs, kidneys, and brain (Cherukuri et al., 2006; Deng et al., 2007; Liu et al., 2007; Georgin et al., 2009; Krug et al., 2011). For example, smaller MWCNTs have been found to distribute more widely throughout the body compared to larger ones (Shvedova et al., 2012). It has been reported that the intravenous administration of MWCNTs was safe with very low toxicity. Yang et al. investigated the long-term effects of intravenously administered MWCNTs in mice and found that the nanotubes accumulated in the lungs, liver, and spleen, with minimal inflammatory cell infiltration in the lungs (Yang et al., 2008). Similarly, Schipper et al. conducted a pilot study in mice and reported no observable toxicity when MWCNTs were administered intravenously. These findings suggest that intravenous administration of MWCNTs is relatively safe and may be a promising method for drug delivery and other applications (Schipper et al., 2008).

The biodistribution of MWCNTs in mice was determined by using the skeleton 13C-enriched SWNTs and isotope ratio mass spectroscopy. The study found that there were no acute toxic effects observed in animals exposed to high doses of 13C-SWNTs, and no animals died during the 4-week test period. However, the clearance rate of the nanotubes from most organs was slow, with significant accumulations in the liver, lungs, and spleen (Yang et al., 2007). In another study, the long-term toxicity of MWCNTs in mice after intravenous exposure was investigated. It was found that the MWCNTs accumulated in various organs, including the liver, spleen, and lungs, for up to 3 months post-exposure (Deng et al., 2008). Similarly, the biodistribution of radio labelled MWCNTs in mice using *in vivo* positron emission tomography was investigated. The study found that MWCNTs functionalized with phospholipids bearing PEG were stable *in vivo*, and PEG chain length affected their biodistribution and circulation. MWCNTs coated with PEG chains linked to RGD peptide efficiently targeted integrin-positive tumors in mice, exhibiting high tumor accumulation due to the multivalent effect of the MWCNTs. The MWCNTs were largely localized in the liver and lesser localized in spleen and kidney. The PEG caused a slower blood clearance and lower concentration in the liver (Liu et al., 2007).

The distribution of 14C-taurine-MWCNT after intravenous injection was investigated in mice. Results showed that 75% of the dose accumulated in the liver after 1 month but decreased to 20% after 90 days. MWCNT were observed in Kupffer cells via TEM imaging, with <5% found in the spleen and lungs which are almost eliminated in 90 days (Deng et al., 2007). The same group of researchers tested biodistribution of two types of functionalized MWCNTs, 125I-Taurine-MWCNTs, and 125I-Tween-80-MWCNTs by intravenous administration into mice. About 75% of 125I-tau-MWCNT was in the liver up to 6 h, very small amount was seen in other organs such as spleen lungs. 125I-Tween-MWCNT was found to be distributed in many organs, liver, spleen and lung, stomach, kidney, large and small intestine, probably due to amphiphilic properties of Tween 80 (Deng et al., 2008). The biodistribution of 14C-MWCNT suspension (in rat serum) after intravenous administration was followed in rats. The MWCNTs were cleared from the bloodstream and were predominantly distributed to the liver. The lungs, spleen, and kidneys also had lower amounts, whereas no radioactivity was found in the brain, heart, bones, stomach, and muscles. Dark clusters were observed in the lungs and liver via optical microscopy, which coincided with radioactive hotspots. Throughout the study, radioactivity in all organs decreased (Deng et al., 2008; Mortensen et al., 2022). The impact of MWCNTs of two different diameters on the biodistribution was followed. The MWCNTs were functionalized and conjugated with radionuclide chelating moieties and IgG antibodies and a radioactive tracer. The results showed that the modification did not affect the distribution but the narrow MWCNTs (9 nm) had less tissue affinity than wider MWCNTs (40 nm) suggesting their suitability of biological application (Singh et al., 2006).

In a recent study, the influence of the diameter and length MWCNTs on biodistribution was investigated. Three different sized carboxylated MWCNTs were administered as a single intravenous dose of 1 mg/kg MWCNTs. Biodistribution was evaluated in liver, lung, spleen, and lymph nodes using microscopy and hyperspectral imaging. The results showed that the observed tissue location of MWCNTs is size dependent. Overlaps in the perturbation of endogenous metabolite profiles were found regardless of their size. The tissue distribution and persistence of MWCNTs in liver, spleen, lymph node, and lung are influenced by their size. The metabolomics findings suggest that the liver is impacted by the MWCNTs observed in the tissue. Water-soluble MWCNTs functionalized with DTPA and labeled with indium were used for urine excretion studies in rats. Imaging showed that within a minute, the CNT began to accumulate in the kidneys and bladder. At 30 min, most of the detected activity was in the kidneys/bladder. At 6 h, almost all CNT eliminated via renal excretion route. Urinary excretion of the vast majority of radiolabeling (nanotubes) was confirmed at 24 h, where it was shown that 11.5% of the dose/g tissue was in the urine, whereas the liver, spleen, bladder, and kidneys all had content below 1% (Singh et al., 2006; Deng et al., 2008; Mortensen et al., 2022). While the biodistribution of CNTs and qualitative assessments of CNT deposition in tissues have been well reported in the literature, the quantification of CNTs at this point is technically demanding.

Factors affecting the distribution and elimination of MWCNTs:

MWCNTs are distributed throughout the body and eliminated. Various factors, including size, shape, and surface functionalization have been shown influence the absorption, distribution, metabolism, and excretion of MWCNTs, potentially impacting their pharmacokinetics and toxicity (Liu et al., 2013; Mehra et al., 2015; Mortensen et al., 2022).

• MWCNTs that are smaller in size have the potential to penetrate tissue more deeply and may be eliminated more effectively by renal filtration. Larger MWCNTs on the other hand accumulate in the spleen and liver, among other organs.
• Variations in the shapes of MWCNTs, such as straight, curved, or tangled forms, can affect cellular uptake and their interaction with biological membranes.
• Surface functionalization of MWCNTs influences circulation time, biodistribution, and clearance routes by altering their interaction with bio molecules and cells.
• The surface charge of MWCNTs influences how they interact with proteins, cells, and tissues, which in turn impacts their biodistribution and clearance from the body.

## 5 Conclusion

Ligand-functionalized MWCNTs have emerged as effective nanocarriers for anticancer drug delivery, owing to their biocompatibility and low toxicity to healthy cells and tissues. A variety of ligand molecules (Table 2) that bind to specific receptors found on cancer cells can be attached to MWCNTs via chemical/biochemical linker moieties, affording significant opportunities for the targeted delivery of anticancer therapeutics in different cancers. Ligand-functionalized MWCNTs have demonstrated increased aqueous dispersibility, cellular uptake, bioavailability, and drug loading capacity, making them useful for tumor-targeted delivery of anticancer drugs with poor pharmacokinetic properties, and poor cell permeability. Furthermore, ligand-functionalized MWCNTs display relatively less toxicity and greater penetrability through biological membranes making them ideal for biological applications. Overall, we conclude that functionalized MWCNTS are unique, multifunctional nanocarriers that hold considerable promise for cancer-targeted drug delivery. The rapid advancement in MWCNT functionalization strategies together with the availability of a wider array of cancer cell targeting ligands would pave the way for the translational development of these novel nanoplatforms for the diagnosis and treatment of cancer.

**TABLE 2:** A summary of the *in vitro* and *in vivo* effect of bioactive targeting ligands conjugated with MWCNTs on cancer cells and tumors.

CNTs	Modification in linker and nanomolecules	Types of ligands	Receptors	Anticancer drugs	Cell line/Targets	Key finding	References
MWCNTs	-	Thiamine/Riboflavin	-	Paclitaxel	MCF-7	Increased tumor cell penetration	(Singh et al., 2016 (a))
MWCNTs	DMTMM, FITC and Peptide	Folate	FAR	Vinca alkaloide	HT-29 CCD841 and Saos-2	Folate, DMTMM, FITC and Peptide Multifunctionalized MWCNTs enhanced the cellular uptake in cancer cells as compared to only folate functionalized MWCNTs	Fraczyk et al. (2017)
MWCNTs	Magneto-fluorescent	Folate	FAR	Doxorubicin	HeLa and MCF-7 cells	Enhanced the chemo-photothermal synergistic therapy by modified MWCNTs with magneto-fluorescent, which is helpful to decreased the growth of tumors	Zhang et al. (2018)
MWCNTs	Poly (N-vinyl pyrrole)	Folate- thiolended polyethylene glycol	FAR	Doxorubicin	HeLa cells	Improved the drug loading, cell viability and targeted drug delivery	Wang et al. (2017)
MWCNTs	Polyethylene glycol	Folate-doxorubicin	FAR	Cisplatin	MCF-7 cells	Enhanced the cellular cytotoxicity by using dual drug combination	Yang et al. (2017)
MWCNTs	Polyethylene glycol bis amine	Folate	FAR	5-fluorouracil	MCF-7	Reduced cell proliferation of MCF-7	Kaur et al. (2017)
MWCNTs	PEGylation	Folate	FAR	Doxorubicin	MCF-7	Improved the bio-distribution and elimination rate of doxorubicin	Mehra et al. (2013)
MWCNTs	Radiotracer and fluorochrome	Folic acid	FAR	Methotrexate	A549 and MCF-7	Enhanced the accumulation of Dox in tumor and decreasing the tumor growth	Das et al. (2013a)
MWCNTs	Polyethylene glycol	Folic acid, Estradiol	FAR	Methotrexate, Doxorubicin, Paclitaxel	A549, HeLa and MCF-7	Functionalized MWCNTs loaded drugs enhanced the efficacious as compared to plain drugs	(Das et al. (2013b)
MWCNTs	-	Folate	FAR	Gemcitabine	MCF-7	Improved the biodistribution and drug loading efficiency of gemcitabine	Singh et al. (2013)
MWCNTs	poly (acrylic acid) Iron-oxide magnetic nanoparticle	Folate-FITC	FAR	Doxorubicin	U87 cells	Enhanced the drug loading efficacy and cell cytotoxicity	Lu et al. (2012)
MWCNTs	Glycine	-	FAR	Methotrexate	MDA-MB-231 cells	Improved the cell viability, drug loading and half-life of methotrexate	Joshi et al. (2017)
MWCNTs	Iron NPs	Folic acid	FAR	Doxorubicin	HeLa cell	Increased the loading efficiency and cell viability	Li et al. (2011)
MWCNTs	poly (amidoamine)dendrimers	Folic acid- FITC	FAR	-	KB cells	Enhanced folate receptor binding and reduce the proliferation of cells	Shi et al. (2009)
MWCNTs	Oridonin loaded liposome containing microbubbles	Folic acid	FAR	Oridonin	HepG2 cancer cell	Enhanced the cellular uptake and decreased tumor growth	Wang et al. (2019)
MWCNTs	Polyethyleneimine, fluorescein isothiocyanate, polyethylene glycol	Folic acid	FAR	Doxorubicin	MCF-7 breast cancer cell	Improved the receptor binding efficacy and tumor growth inhibition	Yan et al. (2018)
MWCNTs	Chitosan	Folic acid	FAR	Docetaxel/Coumarin-6	A549 lung cancer cell	Increased the drug loading and reduces cancer cell proliferation	Singh et al. (2017)
MWCNTs	Polypyrrole and gold nanoparticles	Folic acid	FAR	Doxorubicin	HeLa and H9C2 cancer Cells	Inhibited the proliferation of Hela cells and H9C2 cardiomyoblast cells	Wang et al. (2017)
MWCNTs	poly (1-O-methacryloyl-b-D- fructo-pyranose-b-(2-methacryloxyethoxy)) benzaldehyde	Folic acid	FAR	Doxorubicin	MDA-MB-231 & MCF-7 cells	Enhanced the dispersibility, apoptosis and provided dual targeted effect in breast cancer cells	Ozgen et al. (2020)
MWCNTs	poly-L-lysine, adipic acid	Folic acid	FAR	Doxorubicin	HEK293 & HepG2 cells	Improved cell viability and apoptosis	Zhou et al. (2022)
MWCNTs	-	D-a-tocopheryl polyethylene glycol 1000 succinate	G1 phase	Docetaxel/Coumarin 6	A-549 cells	Enhanced the drug loading, cellular uptake and inhibited the cell proliferation	(Singh et al., 2016 (b))
MWCNTs	-	α-tocopheryl succinate and chondroitin sulphate	CD44 receptor	Doxorubicin	MDA-MB-231 breast cells	Increased cellular uptake, drug loading capacity and provided pH dependent release	Jain et al. (2020)
MWCNTs	Polyethylenimine	Hyaluronic acid- Fluorescein isothiocyanate	CD44 receptor	Doxorubicin	HeLa and L929 cells	Enhanced cell proliferation inhibition rate	(Datir et al., 2012)
MWCNTs	-	Hyaluronic acid	hyaluronan receptor	Doxorubicin	A549 cells	Enhanced cellular uptake and CD44 receptor targeted selectivity	Cao et al. (2015)
MWCNTs	α-Tocopheryl succinate	hyaluronic acid	hyaluronan receptor	Doxorubicin	MDA-MB-231 breast cells	Improved drug loading capacity, cytotoxicity and dispersibility	Singhai et al. (2020)
MWCNTs	-	Gonadotrophin hormone	-	Doxorubicin	DU-145	Increased GnRH receptor binding efficacy	Chen et al. (2002)
MWCNTs	Chitosan	-	-	Doxorubicin	BEL-7402 hepatoma cell	Improve cell proliferation and cell uptake	Dong et al. (2017)
MWCNTs	poly (ethylene glycol)	-	-	Paclitaxel	MCF-7, HeLa cells	Developed sustained releases	Lay et al. (2010)
MWCNTs	cis-Pt (1,7-phenanthroline), ferric oxide, poly (ethylene glycol), poly (citric acid)	-	-	Cisplatin	MDA-MB-231 and HeLa cells	Developed safe and effective multifunctionalized nanocarrier, which is reduces the cell growth	Rezaei et al. (2018)
MWCNTs	Polyethylene glycol	-	-	Curcumin	C6 brain cancer cells	Improve biocompatible and inhibiting brain cancer cell growth n	Allegra et al. (2017)
MWCNTs	Poly (acrylic acid)-poly (ethylene glycol)	-	-	Cyclophosphamide and methotrexate	-	Increased sustained release	Azqhandi et al. (2017)
MWCNTs	Piperine	-	-	Docetaxel	MDA-MB-231 cancer cells	Enhanced cytotoxicity and decreased clearance rate	Raza et al. (2016)
MWCNTs	Chitosan	-	-	5-Fluorouracil	MCF-7 breast cancer cells	Improved loading efficacies of drug and cancer cell proliferation	Nivethaa et al. (2016)
MWCNTs	Polyethylene glycol	-	-	Doxorubicin	HepG2, SH-SY5Y and HT-29 cancer cells	Enhanced dispersibility and drug loading capacity	Pistone et al. (2016)
MWCNTs	biotinylated chitosan	Biotin	tyrosine kinase	neratinib	SkBr3 cell line	enhanced the cytotoxicity as well as provided pH depended on releases	Selim et al. (2023)
MWCNTs	guanidinylated dendritic	-	-	Doxorubicin	HEK293, PC3 and DU145	enhanced the cell cytotoxicity, dispersibility as well as loading capacity	Lyra et al. (2021)
MWCNTs	Chitosan	Galactose	ASGP-R	Doxorubicin	HepG2 cell	Improved the cell cytotoxicity, dispersibility and drug loading capacity	Qi et al. (2015)
MWCNTs	Ethylene-diamine	Galactose	-	-	-	Enhanced the aqueous dispersibility	Jain et al. (2009)
MWCNTs	lysine	galactose, mannose, lactose	Lectin receptor	Doxorubicin	MDAMB231 & MCF7 cells	Improved the MWCNTs dispersibility, enhanced cell cytotoxicit and Dox loading efficiency	Thakur et al. (2022)
MWCNTs	Poly (diallyldimethylammonium chloride)	Lactose-glycine	ASGP-R	-	HEK293, Huh7 cell	Enhanced dispersibility and accumulation rate to the tumor target site	Liu et al. (2014)
MWCNTs	Ethylene-diamine	Fucose	ASGP-R	Sulfasalazine	J774 cell	Decreases cell viability and improved drug loading efficacy	Gupta et al. (2014)
MWCNTs	Ethylene-diamine	Dexamethasone mesylate	Nuclear receptor	Doxorubicin	A-549 cells	Improved dispersibility and cell cytotoxicity	Lodhi et al. (2013)
MWCNTs	Ethylene-diamine	Glycyrrhizin	ASGP-R	Doxorubicin	HepG2 cells	Decreased cancer cell proliferation	Chopdey et al. (2015)

Abbreviations: FAR, folic acid receptor or folate receptors; ASGP-R, asialogycoprotein receptor.
